# The Voronoi Diagram of Rotating Rays with Applications to Floodlight Illumination

**DOI:** 10.1007/s00453-025-01368-y

**Published:** 2026-03-09

**Authors:** Carlos Alegría, Ioannis Mantas, Evanthia Papadopoulou, Marko Savić, Carlos Seara, Martin Suderland

**Affiliations:** 1 Dipartimento di Ingegneria, Università Roma Tre, Rome, Italy; 2 Faculty of Informatics, Università della Svizzera italiana, Lugano, Switzerland; 3 Department of Mathematics and Informatics, Faculty of Sciences, University of Novi Sad, Novi Sad, Serbia; 4 Departament de Matemàtiques, Universitat Politècnica de Catalunya, Barcelona, Spain

**Keywords:** Rotating rays Voronoi diagram, Oriented angular distance, Brocard angle, Brocard illumination, Floodlight illumination, Coverage problems, Theory of computation, Computational geometry

## Abstract

We study the *Voronoi Diagram of Rotating Rays*, a Voronoi structure where the input sites are rays and the distance function between a point and a site/ray is their counterclockwise angular distance. This novel Voronoi diagram is motivated by illumination and coverage problems, where a domain must be covered by floodlights, which are wedges of uniform angle, and the goal is to find the minimum angle necessary to cover the domain. This angle is called the *Brocard angle* and it is encoded in the Voronoi diagram of rotating rays. We study the diagram in the plane and present its structural properties, its combinatorial complexity bounds, and a construction algorithm. If the rays are induced by a convex polygon, we show how to construct the rotating rays Voronoi diagram within the polygon in optimal linear time. We can thus compute the Brocard angle of the polygon in the same time. Floodlights are used to model devices with limited sensing range, like surveillance cameras or directional antennas. In this context, the Brocard angle reveals the minimum range necessary for a set of devices to cover a domain.

## Introduction

**Fig. 1:**
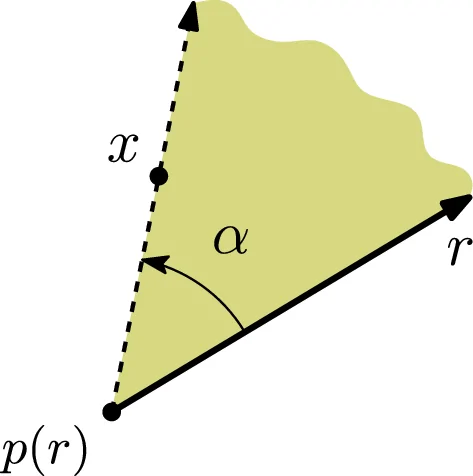
An *α *-floodlight aligned with a ray *r* with apex *p*(*r*). The angle *α * is the angular distance from *r* to the point *x*

**Fig. 2:**
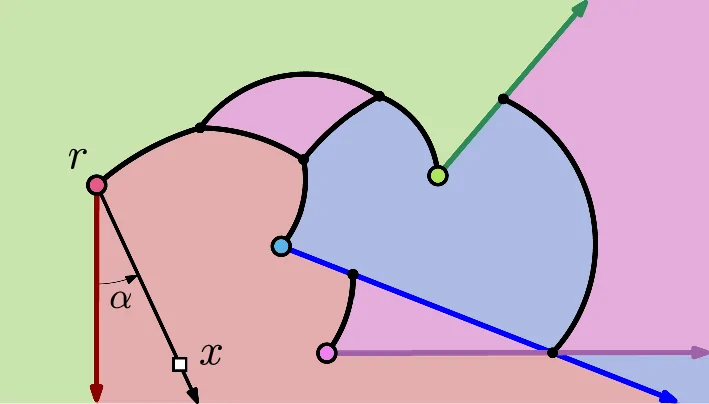
The rotating rays Voronoi diagram of 4 rays in $\mathbb {R}^2$. The points in each region are first illuminated by the ray of the respective color. The angle *α * is the distance of the point $x \in \mathbb {R}^2$ to its nearest site (ray *r*)

In this paper, we formulate the *rotating rays Voronoi diagram*, which is defined by a set of *n* rays in the plane under the following oriented angular distance function. Given a point *x* and a ray *r* in the plane, the *angular distance* from *r* to *x* is the smallest angle *α * such that, after a counterclockwise rotation of *r* around its apex by *α *, the ray *r* *illuminates* (or touches) *x*; see Fig. 1. An example diagram is illustrated in Fig. 2. We define the rotating rays Voronoi diagram, present related combinatorial properties and bounds, design construction algorithms, and use the diagram to solve related floodlight illumination problems.

**Motivation**. Floodlight illumination problems are well known *art gallery* type of problems, where a given domain has to be covered by *floodlights*, which are light sources illuminating the interior of a cone from its apex. A floodlight of aperture *α * is called an *α **-floodlight*. An *α *-floodlight is said to be *aligned with* a ray *r*, if the right side of the floodlight coincides with *r*; see for example Fig. 1.

The angular distance considered in this work is motivated by the *Brocard illumination problem*: Given a domain $\mathcal{D}$, a set $\mathcal{S}$ of *n* rays, and a set of *n* *α *-floodlights each aligned with a ray of $\mathcal{S}$, what is the minimum angle $\alpha ^*$ required to illuminate $\mathcal{D}$ with the set of $\alpha ^*$-floodlights? The angle $\alpha ^*$ is called the *Brocard angle* of $\mathcal{D}$.

**Fig. 3:**
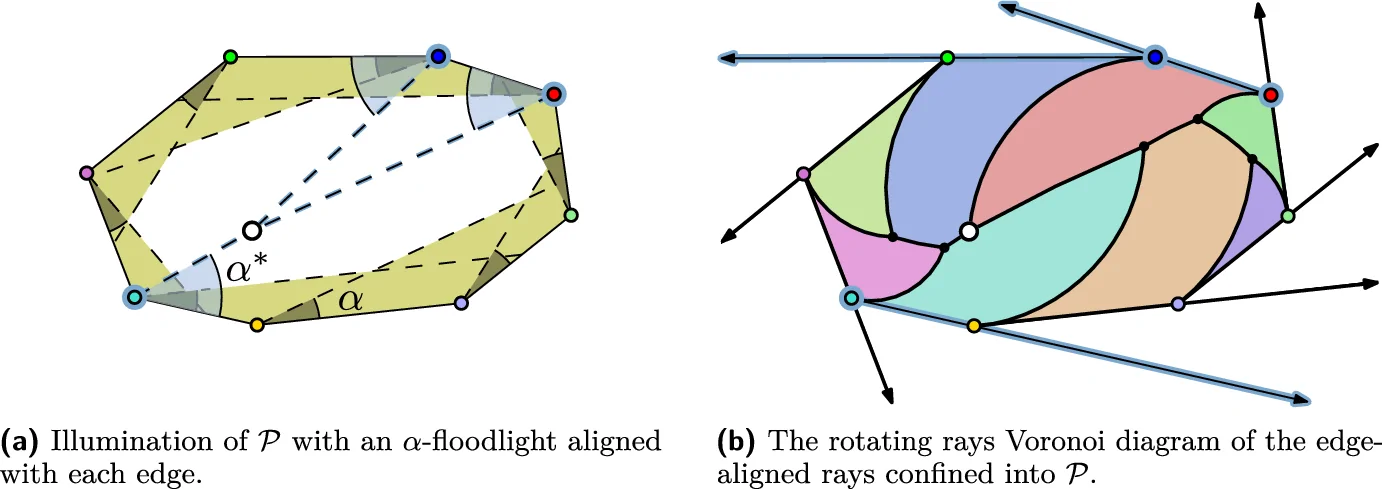
A convex polygon $\mathcal{P}$. Highlighted in blue, the three rays that realize the Brocard angle $\alpha ^*$. The interior point of $\mathcal{P}$ at which $\alpha ^*$ is realized is a rotating rays Voronoi diagram vertex

We show that the Brocard illumination problem can be reduced to constructing the rotating rays Voronoi diagram of $\mathcal{S}$ within $\mathcal{D}$. The reduction is based on the fact that the Brocard angle is realized at a vertex of the rotating rays Voronoi diagram. Typical domains to illuminate by floodlights include the plane, bounded polygonal regions, and unbounded regions such as wedges and curves. See an example of a convex polygonal region in Fig. 3. Since the construction of the respective Voronoi diagram restricted to each domain yields the Brocard angle, there is an interest in studying the Voronoi diagram in different settings.

**Background and related work: Brocard Illumination**. The Brocard illumination problem combines floodlight illumination with a generalization of a classic geometric problem first solved by Henri Brocard (1845-1922). In this problem, the input domain is bounded by a triangle, there is a ray aligned with each side of the triangle, and all the rays are oriented either in clockwise or in counterclockwise direction along the boundary of the triangle. Throughout the years, researchers generalized Brocard’s problem to input domains bounded first by convex quadrilaterals and later by arbitrary convex polygons; see Fig. 3a. A seminal problem derives from a particular class of convex polygons known as *Brocard polygons* [6]: A polygon $\mathcal{P}$ is called a Brocard polygon, if there exists an interior point of $\mathcal{P}$ with equal angular distance to all the rays aligned with the edges of $\mathcal{P}$. The angular distance is precisely the Brocard angle of $\mathcal{P}$, and the point is known as the *Brocard point* of $\mathcal{P}$. Intuitively speaking, the Brocard point of $\mathcal{P}$, if it exists, is the first point simultaneously illuminated by all the *α *-floodlights when we increase the value of *α *, starting at *α = 0*.

The characterization of Brocard polygons has a long history, yet, only harmonic polygons (which include triangles and regular polygons) are known to be Brocard polygons [10]. The classic literature on Brocard polygons studies the Brocard problem from a geometric point of view, yet not from a computational perspective. Nevertheless, from well-known geometric results it is not hard to conclude the following: Given a convex polygon $\mathcal{P}$ with *n* vertices, we can decide whether $\mathcal{P}$ is a Brocard polygon in *O*(*n*) time and, in the affirmative, we can compute the Brocard angle of $\mathcal{P}$ in *O*(1) time.

A natural direction is to consider not only the problem of deciding whether a polygon is Brocard, but the more general problem of computing the Brocard angle of any given polygon. The problem of computing the Brocard angle of a simple polygon has been recently studied by Alegría et al. [2]. The authors gave an $O(n^3\log ^2n)$-time algorithm, and complemented this result with an *O(nłog n)*-time algorithm for convex polygons^1^. To the best of our knowledge, there are no other studies of the Brocard problem from a computational perspective.

**Background and related work: Floodlight illumination**. Since their introduction, floodlight illumination problems have been studied in various settings; refer to book chapters by Urrutia [43] and O’Rourke [32] for surveys of known results. Indicatively, the domain may be the entire plane [8, 13, 37], an unbounded planar region [9, 37], a curve [12, 15, 23, 41], or a polygonal domain [17, 23, 31, 40]. The case when floodlights are required to be of uniform angle, as in the Brocard illumination setting, has been explored by several authors, see for example [11, 17, 22, 29, 33, 40, 42]. From a practical point of view, rotating *α *-floodlights can also be used to model devices with limited sensing range (*field of view*), like surveillance cameras or directional antennas; see for example [5, 27, 28, 39]. In this context, the Brocard angle is interpreted as the minimum range needed for a set of such devices to cover a domain.

**Background and related work: Voronoi diagrams**. Voronoi diagrams are well-studied objects in Computational Geometry with numerous variations and applications; refer to the books of Aurenhammer et al. [4] and Okabe et al. [30] for a comprehensive list of results.

The rotating rays Voronoi diagram is novel with respect to both the input sites and the underlying distance function. Still, in the literature we can find diagrams that explore similar ideas. Motivated by the study of *dominance regions* of players in the analysis of soccer matches [38], De Berg et al. [14] formulated a Voronoi diagram that considers a bidirectional angular distance among rays. More recently, this diagram has also been considered by Haverkort and Klein [20], who formally described the structure of its bisecting curves. The rotating rays Voronoi diagram should not be confused with *visibility-constrained Voronoi diagrams*, in which opaque obstacles, such as rays, may block the visibility of the set of sites [4]. For example, in the Voronoi diagram with visual restrictions [18], the sites are points in the Euclidean plane whose dominance region is restricted to a visibility region bounded by two rays. These rays, however, are only visibility obstacles, and the resulting diagram is not otherwise related to the rotating rays Voronoi diagram.

**Our contribution**. We introduce the *rotating rays Voronoi diagram* and prove a series of results, paving the way for future work on similar problems. We define the rotating rays Voronoi diagram in different domains, and show how the Brocard illumination problem in each domain can be reduced to constructing the corresponding rotating rays Voronoi diagram. More specifically:

- We first consider the diagram of a set of *n* rays in the plane, and identify structural properties which we complement with complexity results: an $\Omega (n^2)$ worst case lower bound and an $O(n^{2+\epsilon })$ upper bound. We also obtain an $O(n^{2+\epsilon })$-time construction algorithm, which we use to find the Brocard angle of the plane induced by the set of rays.
- Motivated by the Brocard illumination problem, we study the diagram in a convex polygonal region bounded by the input set of *n* rays. We present a construction algorithm that runs in optimal *Θ (n)* time, and use this result to find the Brocard angle of a convex polygon in optimal *Θ (n)* time, improving upon the previously known *O(n łog n)*-time algorithm.
  We also discuss the implications of this result to the classic problem of finding the minimum angle $\alpha ^*$, such that three floodlights of $\alpha ^*$-angle can illuminate the entire interior of a convex polygon. We show that, for polygons with at most six vertices, our algorithm improves upon existing results.
- Finally, we study the diagram restricted to simple curves and give a generic construction approach. The combinatorial and time complexity bounds depend on the properties of the particular curve.

**Paper outline**. The rest of the paper is organized as follows. In Sect. 2 we give the necessary preliminaries. In Sect. 3 the domain of interest is the entire plane. In Sect. 4 we consider a convex polygonal domain, and in Sect. 5 the domain of interest is restricted to curves. Section 6 concludes the paper and poses some open questions.

## Preliminaries

Let $\mathcal{S}$ be a set of *n* rays in the plane. Given a ray *r*, we denote its apex by *p(r)*, its supporting line by *l*(*r*), and its direction in $S^1$ by $\widehat{d}(r)$. Given three points $A,B,C \in \mathbb {R}^2$, let *∠ (A,B,C)* denote the counterclockwise angle from $\overrightarrow{BA}$ to $\overrightarrow{BC}$, where $\overrightarrow{BA}$ denotes the ray with apex *B* passing through *A*, and $\overrightarrow{BC}$ respectively. We define the distance function as follows.

### Definition 1

Given a ray *r* and a point $x \in \mathbb {R}^2$, the *oriented angular distance* from *x* to *r*, denoted by $d_{\angle }(x,r)$, is the minimum counterclockwise angle *α * from *r* to a ray with apex *p(r)* passing through *x*; see Fig. 1. Further, we define $d_{\angle }(p(r),r)=0$.

It is easy to see that the oriented angular distance (or angular distance, for short) is not a metric. Moreover, $d_{\angle }(x,r)$ takes values in *[0,2π )* and there is a discontinuity at *2π *. Using this distance function, we can define the bisector of two rays.

### Definition 2

Given two rays *r* and *s*, the *dominance region* of *r* over *s*, denoted by *dr(r,s)*, is the locus of points with smaller angular distance to *r* than to *s*, i.e.,

$$\begin{aligned} & dr(r,s) := \{\, x \in \mathbb {R}^2 \mid d_{\angle }(x,r) < d_{\angle }(x,s) \,\}. \end{aligned}$$

**Fig. 4:**
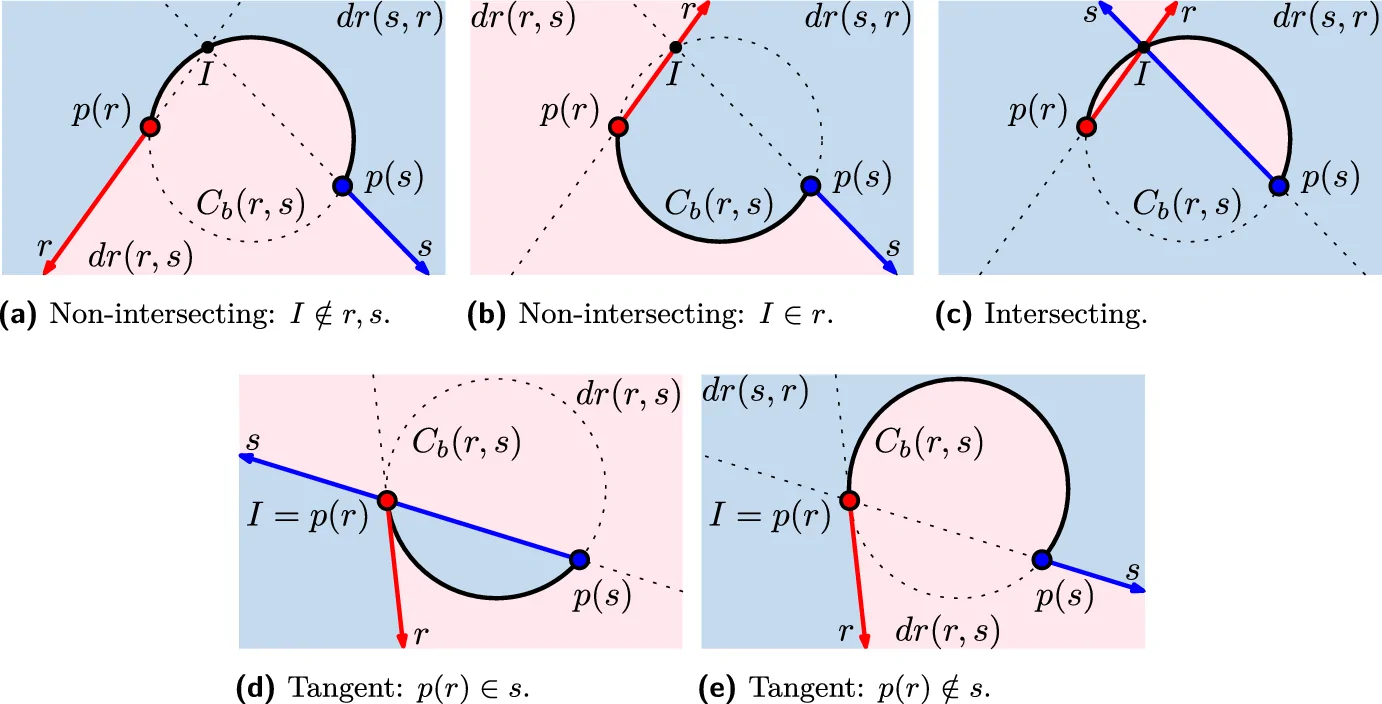
The angular bisector of two rays *r* and *s*. The bisector consists of *r* (red ray), *s* (blue ray), and an arc (black curve) of the bisecting circle $C_{b}(r,s)$

**Fig. 5:**
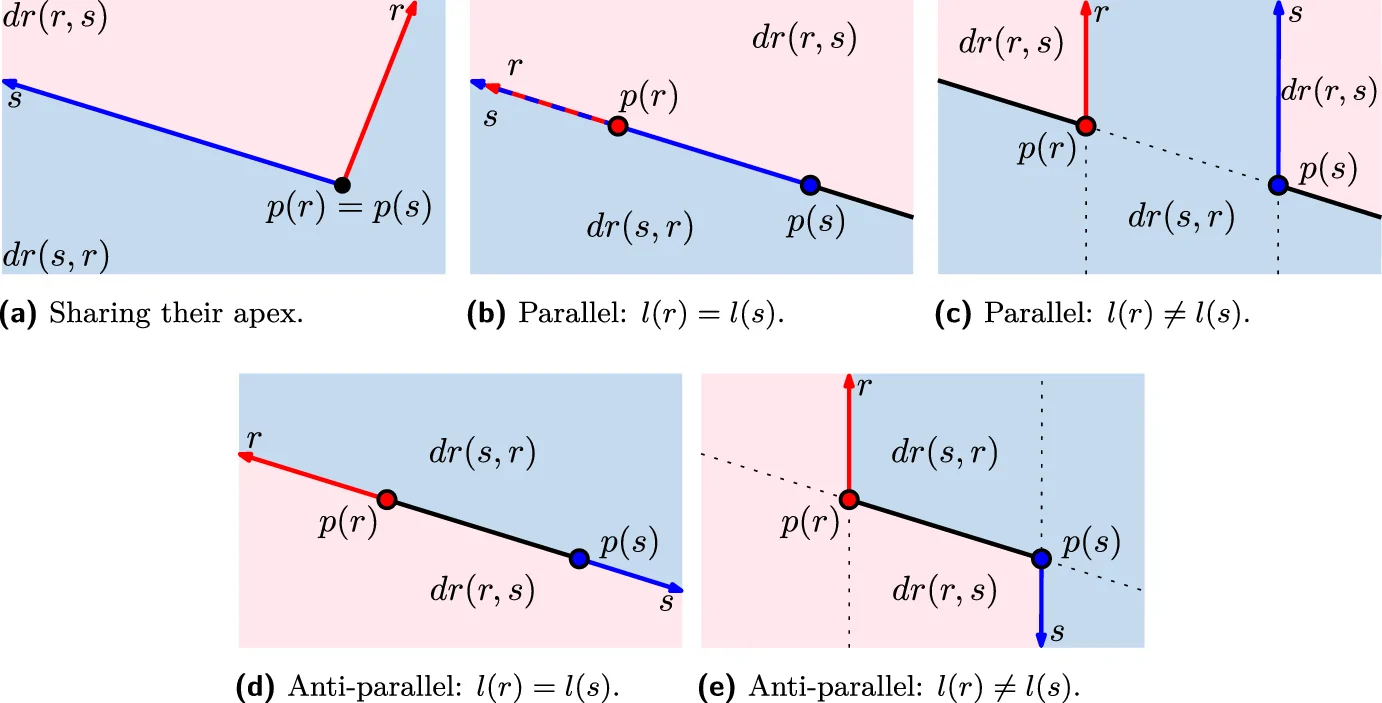
The angular bisector of two rays *r* and *s* (degenerate cases). The bisector consists of *r*, *s*, and a subset (in black) of the degenerate bisecting circle $C_{b}(r,s)$. The bisecting circle degenerates **a** to a point, and **b**–**e** to a line through *p(r)* and *p(s)*

The *angular bisector* of *r* and *s*, denoted by $b_{\angle }(r,s)$, is the boundary of the dominance regions *dr(r,s)* and *dr(s,r)*; see Figs. 4 and 5.

Note that, as a consequence of the discontinuity of the distance function, our definition of a bisector is slightly different from the usual one, which is the locus of points equidistant to two sites.

In Figs. 4 and 5 we show the angular bisector of two rays *r* and *s*. The bisector $b_{\angle }(r,s)$ consists of *r* and *s*, and a (possibly degenerate) circular arc *a* that connects *p(r)* to *p(s)*. The arc *a* belongs to the *bisecting circle* $C_{b}(r,s)$, which is defined depending on the configuration of *r* and *s*. If *l*(*r*) and *l*(*s*) intersect each other in a point *I:= l(r) ∩ l(s)*, we have the following configurations:

- *Intersecting and non-intersecting*. In these configurations, the points *I*, *p(r)*, and *p(s)* are pairwise different. The bisecting circle $C_{b}(r,s)$ is precisely the circle through *I*, *p(r)*, and *p(s)*. In the *non-intersecting* configuration, the rays *r* and *s* do not intersect each other, and the arc *a* contains *I* if and only if *I* lies on none of *r* and *s*; see Fig. 4a and b. Instead, in the *intersecting* configuration, the rays *r* and *s* intersect each other in a point that is different from both *p(r)* and *p(s)*, and the arc *a* contains *I*; see Fig. 4c.
- *Tangent*. In this configuration, the point *I* is equal to either *p(r)* or *p(s)*; see Fig. 4d and e. If *I* is equal to *p(r)*, then $C_{b}(r,s)$ is the circle tangent to *l*(*r*) passing through *p(r)* and *p(s)*. Both *a* and *r* lie on the same side of *l*(*s*) if and only if *p*(*r*) lies on *s*. We analogously define $C_{b}(r,s)$ when *I* is equal to *p(s)*.
- *Sharing their apex*. In this configuration *p(r)* is equal to *p(s)*; see Fig. 5a. Both $C_{b}(r,s)$ and *a* degenerate to a single point.

If instead *l*(*r*) and *l*(*s*) are parallel to each other, we have the following configurations:

- *Parallel*. In this configuration $\widehat{d}(r) = \widehat{d}(s)$; see Fig. 5b and c. The circle $C_{b}(r,s)$ degenerates to the line through *p(r)* and *p(s)*, and *a* consists of one, if *l(r) = l(s)*, or two, if *l(r) ≠ l(s)*, half-lines incident to the respective apices *p(r)* and *p(s)*.
- *Anti-parallel*. In this configuration $\widehat{d}(r) = -\widehat{d}(s)$; see Fig. 5d and e. The circle $C_{b}(r,s)$ degenerates again to the line through *p(r)* and *p(s)*, and *a* degenerates to the line segment connecting *p(r)* to *p(s)*.

Unless otherwise stated, we assume for simplicity that no two rays share an apex and no two rays are parallel or anti-parallel. Under these assumptions, the bisectors are of the forms illustrated in Fig. 4d and e. The assumptions make the presentation of this paper easier, but are not essential for most statements. For instance, Lemma 3 still holds true in projective geometry even for the cases Fig. 5a–e. Here the “circular arc” is part of a circle of infinite radius, and the two segments of Fig. 5c are connected through a point *I* at infinity, i.e., the “intersection” between the two parallel supporting lines. In the following lemma we establish the correctness of the description of the bisectors given above.

### Lemma 3

Given two rays *r* and *s*, the bisector $b_{\angle }(r,s)$ consists of the two rays *r*, *s* and an arc of the bisecting circle $C_{b}(r,s)$.

### Proof

Any point slightly to the left of a ray has a distance of near 0 to this ray, whereas any point slightly to the right of a ray has a distance of almost *2 π *. Hence the rays *r* and *s* are part of the bisector $b_{\angle }(r,s)$.

**Fig. 6:**
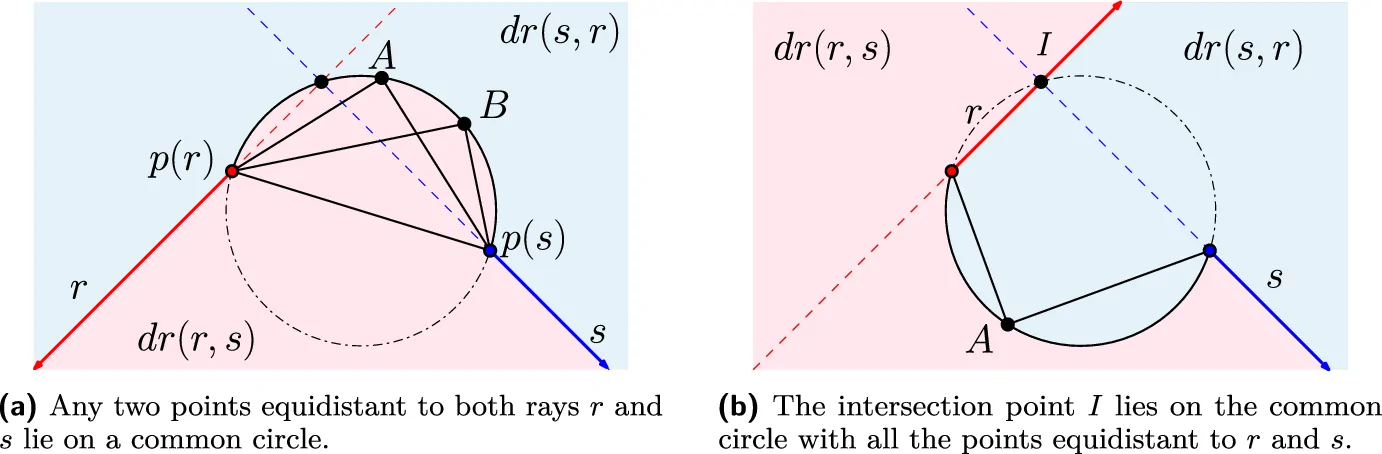
Illustrations for the proof of Lemma 3

We next prove that an arc of $b_{\angle }(r,s)$ belongs to the angular bisector of *r* and *s*. We first show that any two points *A* and *B* equidistant to both rays *r* and *s* lie on a common circle; see Fig. 6a. Since *A*, *B* are equidistant to *r* and *s*, it means that $\angle (B,p(r),A) = \angle (B,p(s),A)$. We show that $\angle (p(r),A,p(s)) = \angle (p(r),B,p(s))$, which implies that all *p*(*r*), *p*(*s*), *A* and *B* lie on a circular arc connecting *p*(*r*) and *p*(*s*) by the inscribed angle theorem:

$$\begin{aligned}&\angle (p(r),A,p(s)) \\ &\quad= \pi - \angle (p(s),p(r),A) - \angle (A,p(s),p(r))\\ &\quad= \pi -(\angle (p(s),p(r),B)+\angle (B,p(r),A))- (\angle (B,p(s),p(r))-\angle (B,p(s),A))\\ &\quad= \pi - \angle (p(s),p(r),B) - \angle (B,p(s),p(r))\\ &\quad= \angle (p(r),B,p(s)). \end{aligned}$$

In the final step we show that *I = l(r) ∩ l(s)* lies on the common circle with all the equidistant points. If *I* lies on both (resp. none) of the rays *r* and *s* then $d_{\angle }(I,r) = d_{\angle }(I,s) = 0$ (resp. $d_{\angle }(I,r) = d_{\angle }(I,s) = \pi$). In this case *I* is equidistant to both rays and therefore, clearly on the common circle.

Let us now assume that *I* lies on exactly one of the rays *r* and *s*; see Fig. 6b. Let *A* be a point equidistant to both rays, i.e., $\angle (I,p(r),A) = \pi + \angle (I,p(s),A)$. Then

$$\begin{aligned} \angle (p(s),A,p(r))&= 2 \pi -\angle (A,p(r),I)-\angle (p(r),I,p(s))-\angle (I,p(s),A)\\&= \pi - \angle (p(r),I,p(s)). \end{aligned}$$

Therefore, by the inscribed angle theorem, *A*, *p*(*r*), *I* and *p*(*s*) lie on opposite sides of a common circle, concluding the proof. *□ *

### Definition 4

The *angular difference* between two rays *r* and *s*, denoted by $\text{ diff}_{\angle }(r,s)$, is the angle by which *s* has to rotate counterclockwise around its apex, so that *r* and *s* become parallel.

**Fig. 7:**
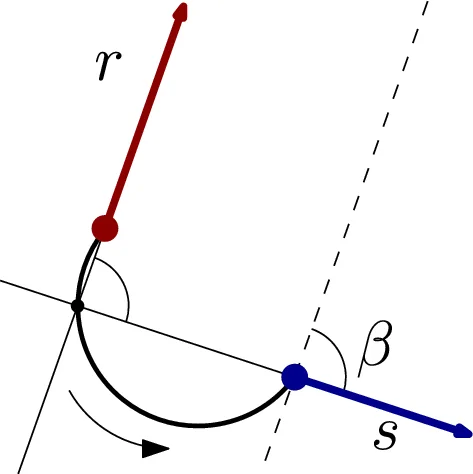
The angular difference $\text{ diff}_{\angle }(r,s) = \beta$. The distance is increasing from *p(r)* to *p(s)* along bisector $b_{\angle }(r,s)$

The angular difference of two rays is illustrated in Fig. 7. Note that for any two non-parallel rays *r* and *s* we have that $\text{ diff}_{\angle }(r,s) + \text{ diff}_{\angle }(s,r) = 2\pi = 0$.

### Remark 5

Given a pair of rays *r* and *s*, the distance function is monotone along the circular arc of their bisector $b_{\angle }(r,s)$, and strictly monotone if the lines *l*(*r*) and *l*(*s*) are not parallel. If the lines *l*(*r*) and *l*(*s*) are parallel, then the distance is constant along the entire circular part of the bisector $b_{\angle }(r,s)$.

If instead $\text{ diff}_{\angle }(r,s) < \text{ diff}_{\angle }(s,r)$, or equivalently $\text{ diff}_{\angle }(s,r) > \pi$, then the distance function along the bisector $b_{\angle }(r,s)$ from *p*(*r*) to *p*(*s*) is monotonically increasing. Moreover, walking along the boundary of *dreg(r,s)* in counterclockwise order, the distance function on the circular part of bisector $b_{\angle }(r,s)$ is monotonically increasing (see the arrow in Fig. 7).

**Fig. 8:**
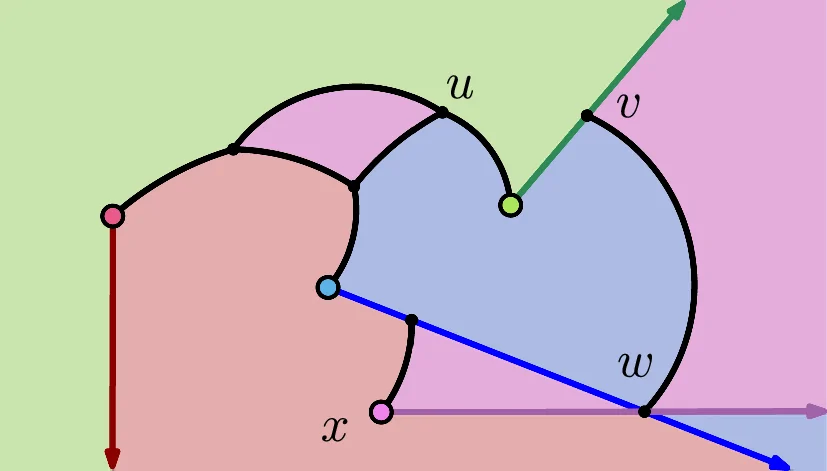
Different features on $\text{ RVD}\!(\mathcal{S})$: arc $\overline{vw}$ is a circular edge, segment $\overline{xw}$ is a ray edge, *u* is a proper vertex, *v* is a mixed vertex, *w* is an intersection vertex, and *x* is an apex vertex

We can now define the nearest Voronoi diagram of a set of rays under the angular distance.

### Definition 6

The *Rotating Rays Voronoi Diagram* of a set $\mathcal{S}$ of rays is the subdivision of $\mathbb {R}^2$ into *Voronoi regions* defined as follows:

$$\begin{aligned} & vreg(r) := \{\, x \in \mathbb {R}^2 \mid \forall s \in \mathcal{S} {\setminus} \{r\}: \ d_{\angle }(x,r) < d_{\angle }(x,s) \, \}. \end{aligned}$$

Let $\text{ RVD}\!(\mathcal{S}):= \left( \mathbb {R}^2 {\setminus} \bigcup _{r \in \mathcal{S}} vreg(r)\right) \cup \mathcal{S}$ denote the graph structure of the diagram.

The Voronoi region *vreg(r)* can be equivalently defined as the intersection of all the dominance regions of *r*, i.e., $vreg(r) = \bigcap _{s \in \mathcal{S} {\setminus} \{r\}} dr(r,s)$. A region may consist of more than one connected components; each component is called a *face* of the region.

$\mathrm{RVD}\!(\mathcal{S})$ has the following two types of edges.

- A *circular edge* is a subset of the circular part of a bisector, thus, any point on a circular edge is equidistant to the two sites that induce it (see $\overline{vw}$ in Fig. 8).
- A *ray edge* is a subset of a ray, thus, any point on a ray edge has distance 0 to the site that induces it (see $\overline{xw}$ in Fig. 8).

Next we consider vertices of the diagram.

- An *apex vertex* is the apex of a ray site. In the diagram it is incident to one circular edge and one ray edge and has distance 0 to the site inducing the ray edge (see *x* in Fig. 8).

We define the following vertices depending on which bisector components intersect, i.e., circular arc – circular arc, circular arc – ray, and ray – ray.

- A *proper vertex* is the intersection of two circular arcs. In fact, it is incident to three circular edges, and is equidistant to the three sites that induce the three circular edges (see *u* in Fig. 8).
- A *mixed vertex* is the intersection of a circular arc with a ray. It is incident to one circular edge and two ray edges, both induced by the same ray. It is equidistant to the two sites inducing the circular edge and has distance 0 to the site inducing the ray edges (see *v* in Fig. 8).
- An *intersection vertex* is the intersection of two rays. It is incident to one circular edge and four ray edges, all of which are induced by two sites. It has distance 0 to both sites (see *w* in Fig. 8).

Note that it is possible for a vertex to fall into several of the above vertex types. For example, consider the apex of one ray lying on top of another ray, as in Fig. 4d. In this case, the apex falls into the apex vertex type as well as the intersection vertex type. These are all possible vertex types in $\mathrm{RVD}\!(\mathcal{S})$ derived by considering the intersections of all possible edge types.

## Rotating Rays Voronoi Diagram in the Plane

In this section we study the diagram $\mathrm{RVD}\!(\mathcal{S})$ in the plane. We first look at some properties and combinatorial complexity bounds. Then we consider the problem of illuminating the plane with a set of floodlights aligned with $\mathcal{S}$.

### Properties, Complexity, and a Construction Algorithm

**Fig. 9:**
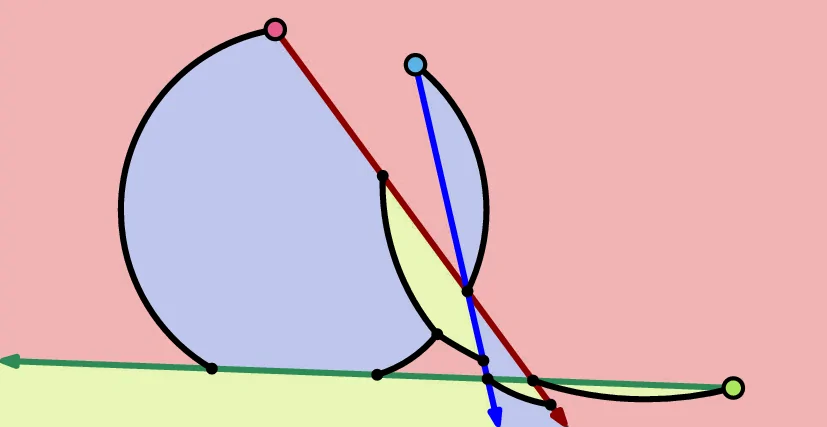
A set $\mathcal{S}$ of 3 rays, with $\mathrm{RVD}\!(\mathcal{S})$ having 1 proper, 5 mixed, and 3 intersection vertices

We first study the structure of the Voronoi diagram of 3 rays; see an example in Fig. 9.

#### Lemma 7

The Voronoi diagram of three rays $\mathrm{RVD}\!(\{r,s,t\})$ has the following vertices: at most 1 proper Voronoi vertex, at most 3 intersection vertices, at most 6 mixed vertices, and exactly 3 apex vertices. Its overall combinatorial complexity is *O*(1).

#### Proof

A proper Voronoi vertex is the intersection point of three circular arcs of three related bisectors. Consider two related bisectors $b_{\angle }(r,s)$ and $b_{\angle }(r,t)$, and their bisecting circles $C_{b}(r,s)$ and $C_{b}(r,t)$. Two circles intersect at most twice, and the circles $C_{b}(r,s)$ and $C_{b}(r,t)$ already have one point of intersection by definition, which is the apex *p(r)*. Hence, there can be at most one more point of intersection *v* between them, and consequently between the circular arcs of $b_{\angle }(r,s)$ and $b_{\angle }(r,t)$. Given an intersection point *v*, the bisector $b_{\angle }(s,t)$ also passes through *v*, inducing a proper vertex of $\text{ RVD}\!(\{r,s,t\})$ at point *v*. So, there exists at most one proper Voronoi vertex in $\text{ RVD}\!(\{r,s,t\})$.

An intersection vertex is defined as the intersection point of two rays. So, given three rays there are at most 3 intersection vertices in $\text{ RVD}\!(\{r,s,t\})$. A mixed vertex is defined at the intersection of a circular arc of a bisector $b_{\angle }(r,s)$ and of a ray $t \notin \{r,s\}$. Consider the circular arc of the $b_{\angle }(r,s)$; the remaining ray *t* can intersect the arc at most two times, hence inducing at most two mixed vertices on the circular arc of $b_{\angle }(r,s)$. Given three rays, there are three bisectors, so overall there can be no more than 6 mixed vertices in $\text{ RVD}\!(\{r,s,t\})$. Finally, each ray has exactly one apex vertex at its apex, so there are exactly 3 apex vertices in $\text{ RVD}\!(\{r,s,t\})$. *□ *

Assuming that no two rays of $\mathcal{S}$ are parallel to each other, the following two simple structural properties hold.

#### Lemma 8

$\mathrm{RVD}\!(\mathcal{S})$ has exactly *n* unbounded faces, one for each ray; each ray is incident to its unbounded face.

#### Proof

**Fig. 10:**
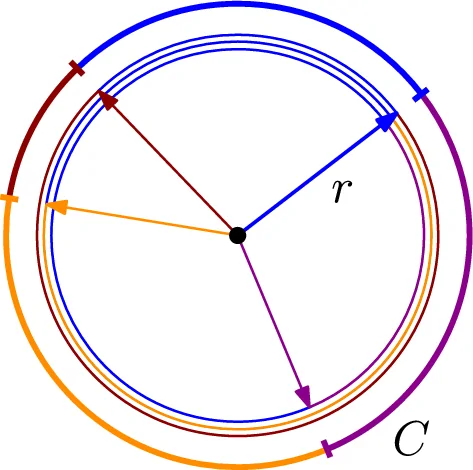
Intersection of a diagram of 4 rays with a large circle *C*. Dominance regions are circular arcs on *C*

Let *C* be a circle of sufficiently large radius so that *C* encloses all vertices of $\mathrm{RVD}\!(\mathcal{S})$ and the bisecting circles of all bisectors. To study the unbounded faces of a Voronoi region *vreg(r)*, for a ray $r\in \mathcal{S}$, we consider the intersection of *vreg(r)* with *C*. Refer to Fig. 10 for an illustration.

Given a ray $s \in \mathcal{S} {\setminus} \{r\}$, the intersection of the dominance region *dr(r, s)* with *C* is a circular arc on *C* lying counterclockwise from *r ∩ C* to *s ∩ C*. Since the region *vreg(r)* is the intersection of all the dominance regions of *r*, it follows that *vreg(r) ∩ C* is the intersection of *n-1* circular arcs, all starting from *r*. Hence, *vreg(r) ∩ C* is a non-empty circular arc, incident to *r*. Thus, *vreg(r)* has exactly one unbounded face incident to *r*. *□ *

**Fig. 11:**
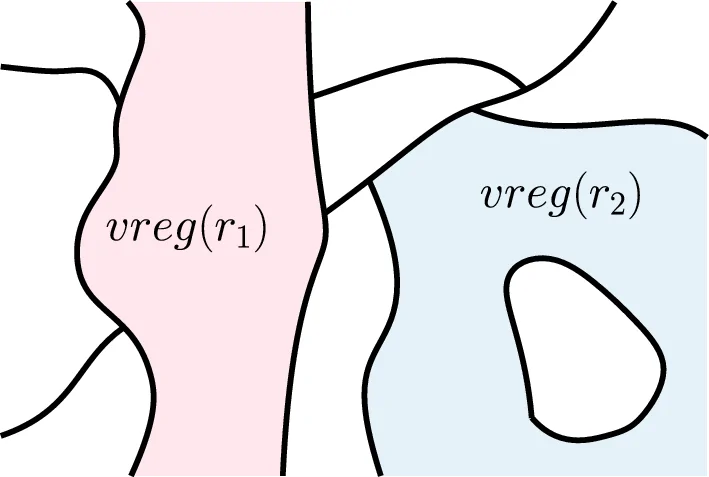
Two (impossible) cases leading to a disconnected diagram. A “corridor” ($vreg(r_1)$) and an “island” ($vreg(r_2)$)

#### Lemma 9

$$\mathrm{RVD}\!(\mathcal{S})$$ is connected.

#### Proof

Assume to the contrary that $\mathrm{RVD}\!(\mathcal{S})$ is not connected, as illustrated in Fig. 11. Then, there exists a ray $r \in \mathcal{S}$ whose region *vreg(r)* either has an unbounded face with two occurrences at infinity forming a *“corridor”* (as $vreg(r_1)$ in Fig. 11), or *vreg(r)* contains a connected component of $\mathrm{RVD}\!(\mathcal{S})$ creating an *“island”* (as $vreg(r_2)$ in Fig. 11).

By Lemma 8, it directly follows that no region of $\mathrm{RVD}\!(\mathcal{S})$ can have a “corridor”. We prove that no region *vreg(r)* of $\mathrm{RVD}\!(\mathcal{S})$ can contain an “island”, i.e., a connected component of $\mathrm{RVD}\!(\mathcal{S})$ entirely surrounded by *vreg(r)*. Suppose to the contrary that there is a component of $\mathrm{RVD}\!(\mathcal{S})$ that is surrounded by *vreg(r)*; this component contains at least one face of a region *vreg(s)* for some $s \in \mathcal{S}, s\ne r$. But then there must be an “island” inside *vreg(r)* in $\mathrm{\!RVD}\!(\{r,s\})$, implying that the bisector $b_{\angle }(r,s)$ has a bounded connected component. This contradicts the fact that each bisector is a single unbounded curve. *□ *

We now study the combinatorial complexity of $\mathrm{RVD}\!(\mathcal{S})$. An $\Omega (n^2)$ lower bound is easily derived by a set $\mathcal{S}$ of *n* pairwise intersecting rays. In such case, $\text{ RVD}\!(\mathcal{S})$ has $$\left( {\begin{array}{c}n\\ 2\end{array}}\right) =\Theta (n^2)$$ vertices (at the intersection of rays) and thus $\Omega (n^2)$ complexity. Interestingly, this bound holds even for non-intersecting rays, as we will show in the following theorem.

#### Theorem 10

The worst case combinatorial complexity of $\mathrm{RVD}\!(\mathcal{S})$ has an $\Omega (n^2)$ lower bound, even if the rays are pairwise non-intersecting.

#### Proof

**Fig. 12:**
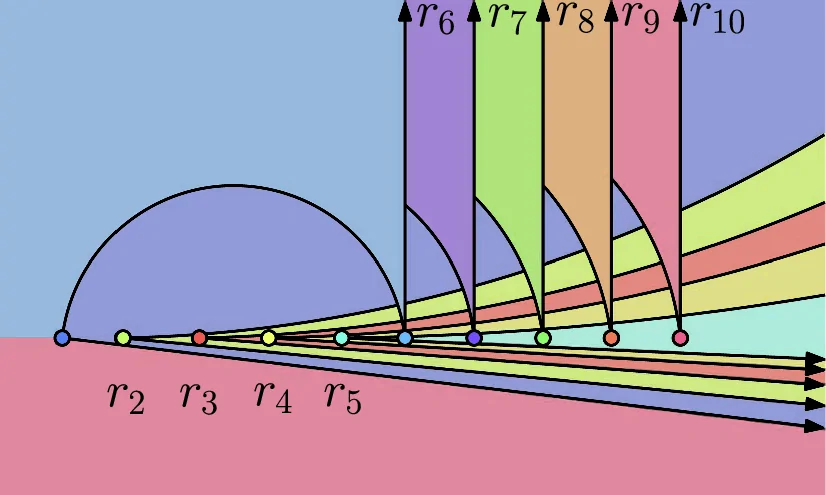
A set $\mathcal{S}$ of *n=10* pairwise non-intersecting rays with $\mathrm{RVD}\!(\mathcal{S})$ having $\Theta (n^2)$ complexity. The region $vreg(r_i)$, *i=1, … , 4*, has *Θ (n)* faces

We give a constructive proof; the resulting diagram is illustrated in Fig. 12. The Voronoi regions of the *n/2 -1* rays with the leftmost apices have *n*/2 bounded faces each.

We set *n = 2m* and let the apices $p(r_i) = (i,0)$, *i=1,…c ,2m*. For *i=m+1,…c ,2m*, let the direction of $r_i$ be vertically upwards. For *i=1,…c ,m*, let the direction of $r_i$ be $\widehat{d}(r_i)=(\sin \alpha _i, \cos \alpha _i)$ with $\alpha _1 \in (3\pi /2,2\pi )$ and $\alpha _i = \alpha _{i-1} + \epsilon _i$ where $\epsilon _i > 0$ for *i=2,…c ,m*. We choose $\epsilon _i$ one by one, in the increasing order of *i*, so that both $r_i$ and $r_{i-1}$ have a face between any two consecutive upward shooting rays. This is always possible since we can choose $\epsilon _i$ small enough so that, at any *x*-coordinate *x < 2m*, the circular part of $b_{\angle }(r_i, r_{i-1})$ is arbitrarily close to the *x*-axis, and thus, is below the circular part of $b_{\angle }(r_{i-2}, r_{i-1})$. *□ *

**Fig. 13:**
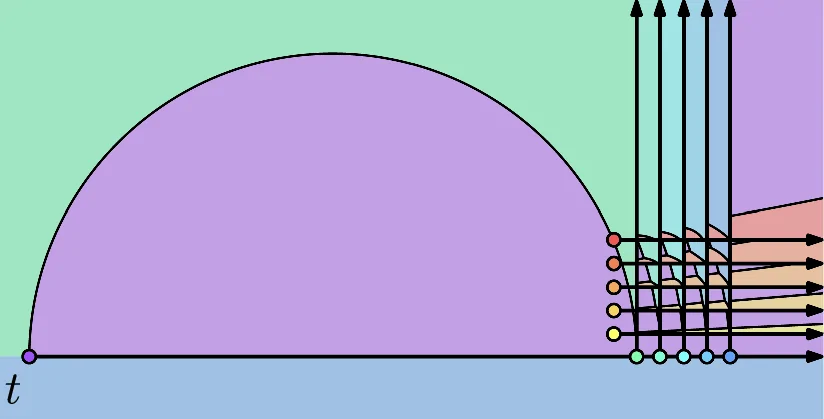
A set $\mathcal{S}$ of *n=11* rays with $\mathrm{RVD}\!(\mathcal{S})$. The region *vreg(t)* has $\Theta (n^2)$ faces, one in each cell of the grid formed by the other 10 rays

#### Theorem 11

A Voronoi region of $\mathrm{RVD}\!(\mathcal{S})$ has $\Theta (n^2)$ complexity in the worst case.

#### Proof

Consider a ray $r \in \mathcal{S}$ and its region *vreg(r)*; all but at most *O*(*n*) vertices (possible apex vertices) on the boundary of *vreg(r)* are defined by *r* and a pair of other sites. There are $\Theta (n^2)$ pairs in $\mathcal{S} {\setminus} \{r\}$, each inducing *O*(1) vertices on *vreg(r)* (by Lemma 7); so *vreg(r)* has $O(n^2)$ vertices, and thus, $O(n^2)$ combinatorial complexity.

We now give a construction of *n=2m+1* rays, where a single region has $\Theta (n^2)$ complexity; refer to the construction shown in Fig. 13. We first create a grid structure: for *i = 1,… ,m*, let $r_i$ be a ray with $p(r_i)= (i,0)$ *shooting* vertically upward and let $s_i$ be a ray with $p(s_i)= (0,i)$ *shooting* horizontally to the right. For all $(i,j) \in \{1, \ldots , m-1\}^2$, let *R*(*i*, *j*) be the square *[i,i+1) × [j,j+1)*. Each square *R*(*i*, *j*) is made up of two faces of $\mathrm{RVD}\!(\{r_1,\ldots ,r_m,s_1,\ldots ,s_m\})$, one belonging to $vreg(r_i)$ and one belonging to $vreg(s_j)$. Now let $\alpha (i,j):= \max \{\, \min \{d_{\angle }(x, r_i), d_{\angle }(x, s_j)\} \mid x \in R(i,j)\,\}$, and let $\alpha _{\min }:= \min \{\, \alpha (i,j) \mid (i,j)$ $\in \{1, \ldots , m-1\}^2\,\}$. It is easy to see that $\alpha _{\min }$ *> \arctan 1/m*.

We now introduce another ray *t*, so that $\max \{\,d_{\angle }(x,t) \mid x \in [1,m]^2\,\} < \alpha _{\min }$. One way to achieve this is to set $p(t) = (-m^2,0)$ and make *t* *shooting* horizontally to the right. This means that in each *R*(*i*, *j*), for $(i,j) \in \{1, \ldots , m-1\}^2$, there is a point which will be visited by the ray *t* before it is visited before any of the rays $r_i$ or $s_j$, meaning the region *vreg(t)* has a face in each square, which is $\Theta (n^2)$ faces in total. *□ *

The above theorem directly implies an $O(n^3)$ upper bound on the complexity of $\text{ RVD}\!(\mathcal{S})$. Next we show how the angular distance function can be adapted so that we can apply the general upper bounds of Sharir [35]. As a by-product, we also obtain a construction algorithm for $\text{ RVD}\!(\mathcal{S})$.

#### Theorem 12

For any *ε > 0*, $\text{ RVD}\!(\mathcal{S})$ has $O(n^{2+\epsilon })$ combinatorial complexity. Further, $\text{ RVD}\!(\mathcal{S})$ can be constructed in $O(n^{2+\epsilon })$ time.

#### Proof

Each site *r* induces a *distance function* $d^r_\angle (x):= d_{\angle }(x,r)$ which maps a point $x = (x_1,x_2) \in \mathbb {R}^2$ to its angular distance from *r*. Consider the lower envelope of the graphs of these distance functions in 3-space. The diagram $\text{ RVD}\!(\mathcal{S})$ can be seen as the projection of this lower envelope to the plane. For algebraic distance functions, Sharir [35] gives complexity bounds for this lower envelope accompanied with algorithmic results. The angular distance functions though are not algebraic. Our strategy is to apply the result from [35] to functions $d^r_{\text {alg}}$ that are equivalent to the functions $d^r_{\angle }$ in the sense that both set of functions would induce the same lower envelope, but each function $d^r_{\text {alg}}$ is made of a constant number of algebraic surface patches in 3-space. More precisely, we want to find piece-wise algebraic functions $d^r_{\text {alg}}$ that fulfill the following property:

$$\begin{aligned} & d^r_\angle (x)<d^s_\angle (x) \, \Longleftrightarrow \, d^r_{\text {alg}}(x) < d^s_{\text {alg}}(x) \end{aligned}$$

for all $r,s \in \mathcal{S}$ and $x \in \mathbb {R}^2$.

Without loss of generality, assume that *p(r)* lies on the origin and *r* is facing to the right in positive $x_1$-direction of the coordinate system. Let $x\in \mathbb {R}^2$ and $\alpha := d^r_\angle (x)$. Then we want to set $d^r_{\text {alg}}(x):= 1 - \cos (\alpha )$ if $0 \le \alpha \le \pi$, and $d^r_{\text {alg}}(x):= 3 + \cos (\alpha )$ if $\pi \le \alpha < 2\pi$. The function $x \mapsto \cos (\alpha )$ is indeed algebraic since it is obtained by first scaling *x* to unit length and then mapping it to its first coordinate. Then we have

$$\begin{aligned} & d^r_{\text {alg}}((x_1,x_2)) = {\left\{ \begin{array}{ll} 0 & \mathrm{if}\quad x_1=x_2=0, \\ 1 - \frac{x_1}{\sqrt{x_1^2+x_2^2}} & \mathrm{if}\quad x_1\ne 0,\, x_2 \ge 0, \\ 3 + \frac{ x_1}{\sqrt{x_1^2+x_2^2}} & \mathrm{otherwise.} \end{array}\right. } \end{aligned}$$

Since $d^r_{\text {alg}}$ consists of three patches, which are all algebraic and have simple domain boundaries, applying [35] to these functions yields the claimed combinatorial and algorithmic results. *□ *

### Brocard Illumination of the Plane

We now look into the Brocard illumination problem in $\mathbb {R}^2$. Recall that given a set of rays $\mathcal{S}$, and an *α *-floodlight aligned with each ray, the problem asks for the Brocard angle which is the minimum angle needed to illuminate a target domain. The Brocard angle of $\mathbb {R}^2$ is

$$\begin{aligned} & \alpha ^*= \max _{x \in \mathbb {R}^2}\min _{r \in \mathcal{S}}d_{\angle }(x,r). \end{aligned}$$

**Fig. 14:**
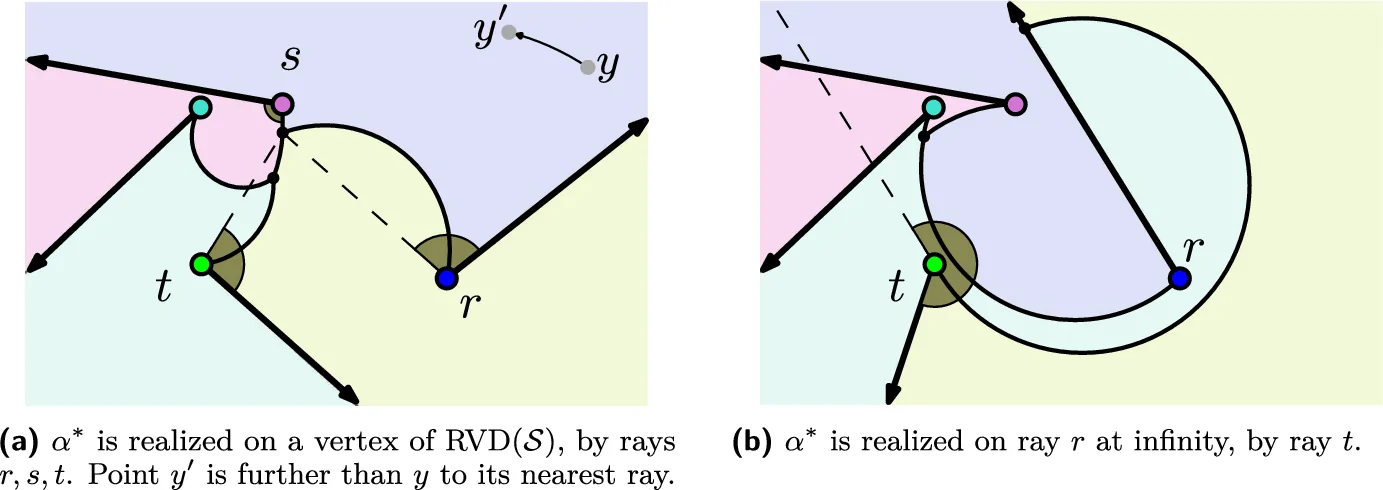
Two examples of the Brocard angle on a set $\mathcal{S}$ of 4 rays in $\mathbb {R}^2$

Let $x^*\in \mathbb {R}^2$ be a point that realizes $\alpha ^*$. Under our assumption that rays in $\mathcal{S}$ are neither parallel nor anti-parallel this point is unique. Then $x^*$ is the last point to be illuminated, assuming that all *n* floodlights start with aperture *α =0* and simultaneously increase their apertures until the entire domain gets illuminated. Although $x^*$ need not be unique, we show that it lies on $\text{ RVD}\!(\mathcal{S})$.

#### Proposition 13

The Brocard angle of a set $\mathcal{S}$ of rays is realized at a vertex of $\text{ RVD}\!(\mathcal{S})$, or at a point at infinity along a ray in $\mathcal{S}$.

#### Proof

We first show that $x^*$ lies on $\text{ RVD}\!(\mathcal{S})$. Suppose, for the sake of contradiction, that $x^*$ does not lie on $\text{ RVD}\!(\mathcal{S})$, but instead, it lies inside the Voronoi region of a ray *r*. Then, we can always find a point with larger angular distance from *r* by simply moving in counterclockwise direction on the circle with center *p(r)* and radius $d(p(r),x^*)$, deriving a contradiction; see for example the points *y* and *y'* in Fig. 14a.

As pointed out in Remark 5, the distance along a circular edge is monotone. Thus, the distance at one of the endpoints of the edge is at least as big as the distance at any point in the interior of the edge. This argument also holds for the distances along ray edges. Hence, a point with maximum distance $x^*$ is either a vertex of $\text{ RVD}\!(\mathcal{S})$ or a point at infinity on a ray of $\mathcal{S}$, concluding the proof. Refer to Fig. 14 for an illustration of the two cases. *□ *

The above implies that we can find $x^*$, and hence $\alpha ^*$, by first constructing $\text{ RVD}\!(\mathcal{S})$ in $O(n^{2+\epsilon })$ time and then traversing the diagram to find the vertex of maximum distance to its nearest neighbors. $\text{ RVD}\!(\mathcal{S})$ is a plane graph, so it can be traversed in linear time using standard techniques. This results in the following.

#### Theorem 14

The Brocard angle of a set $\mathcal{S}$ of *n* rays can be found in $O(n^{2+\epsilon })$ time.

We conclude this section by giving tight bounds on the value of the Brocard angle.

#### Proposition 15

Given a set $\mathcal{S}$ of *n* rays, the range of values of the Brocard angle is $[2\pi /n,2\pi ]$.

#### Proof

**Fig. 15:**
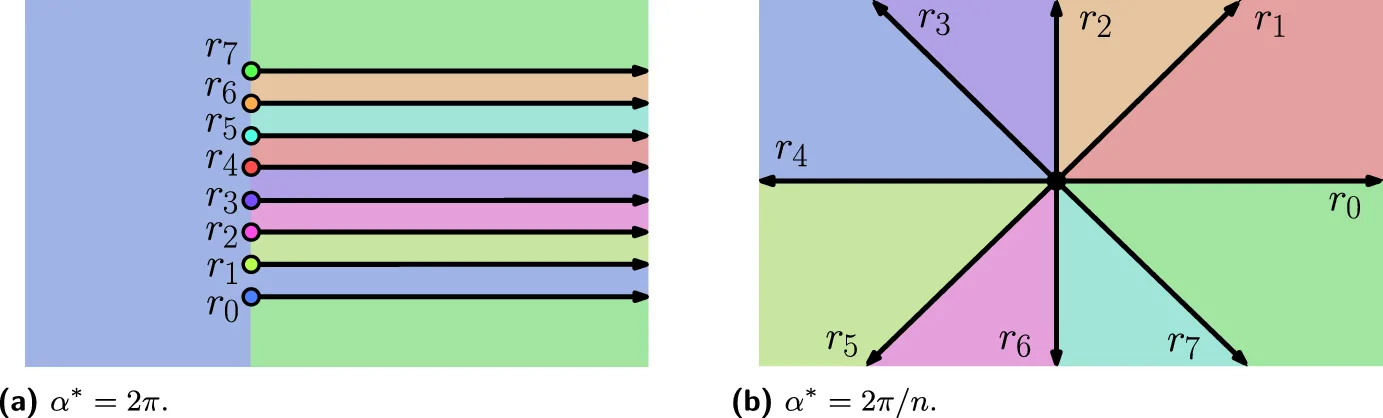
Sets of 8 rays realizing the bounds of the Brocard angle in $\mathbb {R}^2$

For the upper bound, consider a set $\mathcal{S}$ of *n* parallel rays: let $r_i$ have $p(r_i) = (0,i)$ and $\widehat{d}(r_i)=(1,0)$ for $i \in \{0,\dots ,n-1\}$; see the example in Fig. 15a. Observe that the last point to be illuminated is the point on $r_0$ at infinity, i.e., point *(0,+∞ )*, which will be illuminated by $r_{n-1}$ when *α * reaches *2π *; hence the upper bound follows.

For the lower bound, consider that in order to illuminate the entire $\mathbb {R}^2$, all the points *at infinity* should also be illuminated. To illuminate such points, the sum of the angles of all rays should be at least *2π *. Hence, in the best case, a point at infinity is seen by exactly one ray, and a *2π /n* lower bound follows. A construction achieving the *2π /n* bound is the following. Let $\mathcal{S}$ be a set of *n* rays having apex at (0, 0) and with the property that any two consecutive rays have an angular difference of *2π /n*; see the example in Fig. 15b. The last points to be illuminated will be all the points on the right side of each ray $r_i$. These points are illuminated simultaneously by $r_{i-1}$ when *α * reaches *2π /n*. Further, the above construction can be easily adapted to attain any value in $(2\pi /n, 2\pi )$, by expanding a wedge formed by two consecutive rays and shrinking all the others accordingly. *□ *

## Rotating Rays Voronoi Diagram in a Convex Polygon

**Fig. 16:**
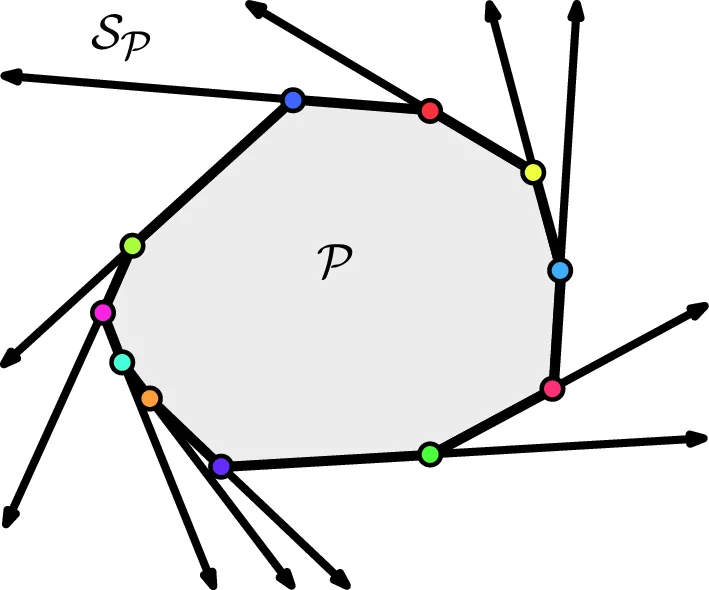
A convex polygon $\mathcal{P}$ and the set of rays $\mathcal{S} _\mathcal{P}$

**Fig. 17:**
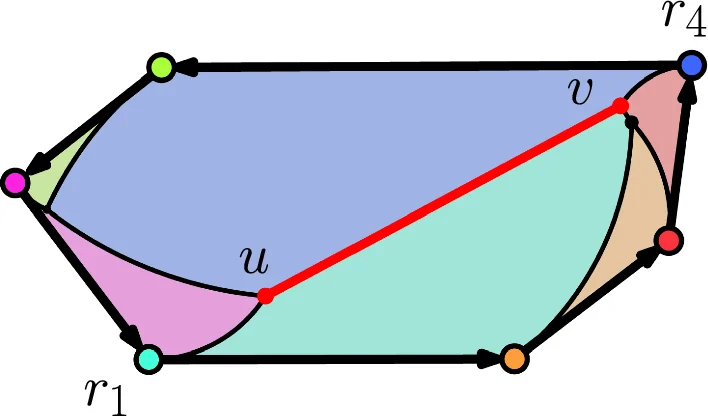
A convex polygon $\mathcal{P}$ with two anti-parallel edges $r_1,r_4$. All points on edge $\overline{uv}$ of $\text{ PRVD}\!(\mathcal{P})$ realize $\alpha ^*$

In this section we present a linear time algorithm to construct the Rotating Rays Voronoi Diagram restricted to the interior of a convex polygonal region. We also show how to use this algorithm to compute the Brocard angle of a convex polygon in optimal linear time. Throughout this section we use the following notation. We denote by $\mathcal{P}$ a convex polygon with *n* vertices, and by $v_1,\ldots ,v_n$ the vertices of $\mathcal{P}$ labeled by appearance while traversing the boundary of $\mathcal{P}$ in counterclockwise direction. For the sake of simplicity, we assume that arithmetic operations on indices are computed using modulo *n*. We denote with $\mathcal{S} _{\mathcal{P}} = \{ r_1,\ldots ,r_n \}$ the set of *n* rays such that the ray $r_i$ leaves the vertex $v_i$ and passes through the vertex $v_{i+1}$, see Fig. 16. Finally, we denote by $\text{ PRVD}\!(\mathcal{P}):= \text{ RVD}\!(\mathcal{S} _{\mathcal{P}}) \cap \mathcal{P}$ the Rotating Rays Voronoi Diagram of $\mathcal{S} _{\mathcal{P}}$ restricted to the interior of $\mathcal{P}$. Examples of this diagram are shown in Figs. 3b and 17.

This section is organized as follows. In Sect. 4.1 we describe a set of basic properties of $\text{ PRVD}\!(\mathcal{P})$. In Sect. 4.2 we describe a simple algorithm to compute $\text{ PRVD}\!(\mathcal{P})$ in *O(nłog n)* time and *O*(*n*) space. Our main result is presented in Sects. 4.3 and 4.5, where we show that $\text{ PRVD}\!(\mathcal{P})$ can be computed in *Θ (n)* time and *O*(*n*) space. Finally, in Sect. 4.6 we discuss the implications of this result in the computation of the Brocard angle of $\mathcal{P}$, and related illumination problems.

### Properties of the Diagram

For the sake of simplicity, throughout this section we make the following assumptions. First, no three vertices of $\mathcal{P}$ are collinear. Second, no point on the boundary or the interior of $\mathcal{P}$ is equidistant to four rays of $\mathcal{S} _{\mathcal{P}}$; this implies that all vertices of $\text{ PRVD}\!(\mathcal{P})$ are incident to at most three edges. Finally, we assume that there are no parallel edges in $\mathcal{P}$, i.e., there are no anti-parallel rays in $\mathcal{S} _\mathcal{P}$. This last assumption guarantees that the Brocard angle is realized at a unique point, which is a vertex of $\text{ PRVD}\!(\mathcal{P})$. Recall from Sect. 2 that the bisector of two anti-parallel rays *r* and *s* contains the line segment connecting *p(r)* and *p(s)*. Therefore, if there are anti-parallel rays, the Brocard angle may be realized on any point of a Voronoi edge, which is part of the bisector of two anti-parallel edges; see an example in Fig. 17.

We start by defining an auxiliary Voronoi diagram, the *Disk Diagram* ($\text{ DD }$), whose system of bisectors consists solely of the set of bisecting circles. This diagram is simpler to study; as we will show, it coincides with the rotating rays Voronoi diagram within $\mathcal{P}$, because its bisectors within the convex polygon $\mathcal{P}$ coincide with the angular bisectors. The disk diagram is formally defined as follows.

#### Definition 16

Given two rays *r* and *s*, the $\text{ DD }$ *dominance region* of *r* over *s*, denoted by $dr_D(r,s)$, is defined as follows:

$$\begin{aligned} & dr_D(r,s) := {\left\{ \begin{array}{ll} \text{the interior of}\, C_{b}(r,s),& \mathrm{if}\quad \mathrm{diff}_{\angle }(r,s) < \pi \\ \text{the exterior of}\, C_{b}(r,s),& \mathrm{if}\quad \mathrm{diff}_{\angle }(r,s) \ge \pi . \end{array}\right. } \end{aligned}$$

The $\text{ DD }$ *bisector* of *r* and *s*, is the boundary of the dominance regions $dr_D(r,s)$ and $dr_D(s,r)$, which is the entire bisecting circle $C_{b}(r,s)$.

#### Definition 17

The *Disk Diagram* of the set $\mathcal{S}$ of rays is the subdivision of $\mathbb {R}^2$ into $\text{ DD }$ *regions* defined as follows:

$$\begin{aligned} & dreg(r) := \bigcap _{s \in \mathcal{S} _\mathcal{P} {\setminus} \{r\}} dr_D(r,s). \end{aligned}$$

Let $\text{ DD }(\mathcal{S})= \mathbb {R}^2{\setminus} \bigcup _{r \in \mathcal{S} _\mathcal{P}} dreg(r)$ denote the graph structure of the diagram.

**Fig. 18:**
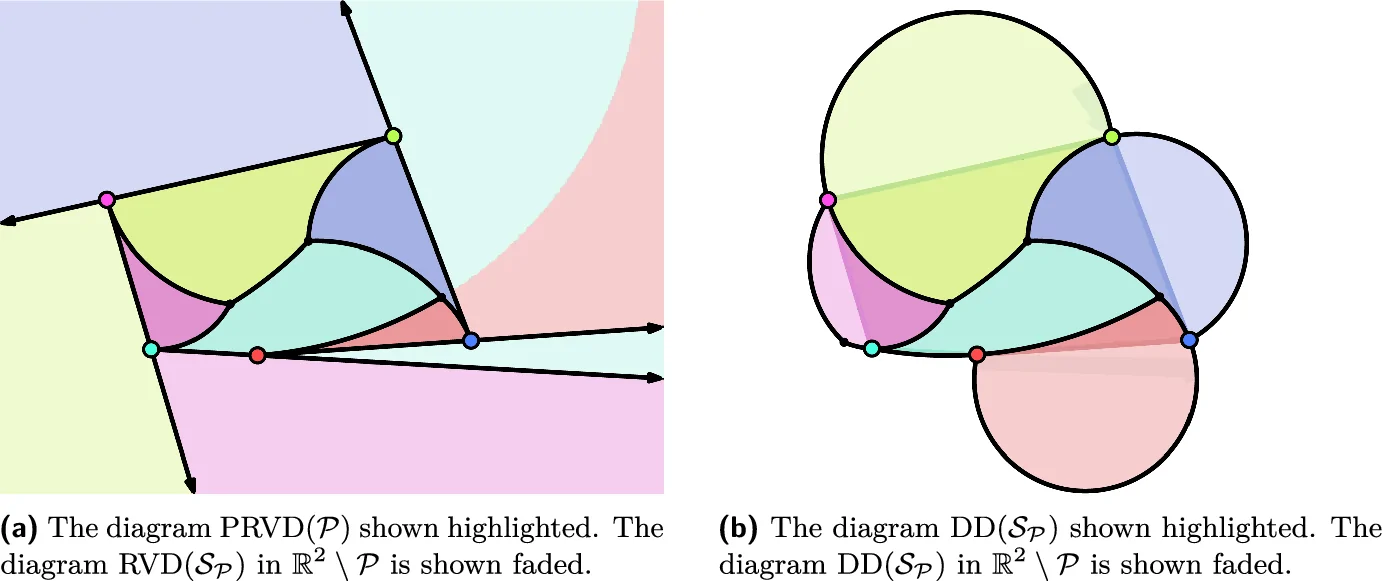
A convex polygon $\mathcal{P}$ with five vertices together with $\text{ PRVD}\!(\mathcal{P})$ and $\text{ DD }(\mathcal{S} _{\mathcal{P}})$

An example of a disk diagram is illustrated in Fig. 18b^2^. Given this definition, every point in the neighborhood of a circular edge in $\text{ RVD}\!(\mathcal{S} _\mathcal{P})$ is associated to the same ray in $\text{ RVD}\!(\mathcal{S} _\mathcal{P})$ and in $\text{ DD }(\mathcal{S} _\mathcal{P})$. Since inside $\mathcal{P}$ the rotating rays Voronoi diagram consists only of circular edges, it follows that $\text{ RVD}\!(\mathcal{S} _\mathcal{P})$ and $\text{ DD }(\mathcal{S} _\mathcal{P})$ are exactly the same in $\mathcal{P}$; see Fig. 18.

#### Lemma 18

The truncated portion of the diagram $\text{ DD }(\mathcal{S} _{\mathcal{P}})$ within $\mathcal{P}$ coincides with $\text{ PRVD}\!(\mathcal{P})$.

#### Proof

It suffices to show that the bisectors of both diagrams coincide within $\mathcal{P}$. Given two rays $r, s \in \mathcal{S} _{\mathcal{P}}$, by Lemma 3 the angular bisector $b_{\angle }(r,s)$ contains the two rays *r*, *s* and a circular arc *α *, subset of the bisecting circle $C_{b}(r,s)$. Since $\mathcal{P}$ is convex, the two rays clearly do not appear in the interior of $\mathcal{P}$, hence we focus on the arc *α *. The DD bisector, by Definition 16, is the complete circle $C_{b}(r,s)$. Thus, we need to show that $C_{b}(r,s) \cap \mathcal{P}$ coincides with *α *.

Let *I* be the intersection point of the supporting lines of *r*, *s*. Due to the convexity of $\mathcal{P}$, point *I* lies outside or on the boundary of $\mathcal{P}$, and only one of *r*, *s* can pass through *I* in their interior. As described in the proof of Lemma 3, this implies that the circular arc of $b_{\angle }(r,s)$ does not pass through *I* in its interior. Thus, the additional circular arc $C_{b}(r,s) {\setminus} \alpha$ does not intersect the interior of $\mathcal{P}$. *□ *

#### Lemma 19

Each region *dreg(r)* of $\text{ DD }(\mathcal{S})$ is connected and contains *p(r)* on its boundary.

#### Proof

By definition, the region *dreg(r)* is formed by the intersection of *n-1* disks or their complements. The boundary of each of these disks is $C_{b}(r,s)$ for some *s*, and since the apex *p(r)* lies on $C_{b}(r,s)$, it also lies on the boundary of the intersection of these disks.

To see that the region *dreg(r)* is connected, we perform an inversion of the plane using *p(r)* as the inversion center, and a circle of arbitrary radius as the inversion circle. This inversion maps circles passing through the inversion center to lines passing through the inversion center, so each dominance region $dr_D(r,s)$ maps to a halfplane. The intersection of halfplanes is connected, and since the inversion preserves connectivity, then region *dreg(r)* is also connected. *□ *

We conclude our detour with the disk diagram as follows; we will use this again in Sect. 4.5. We now turn our attention back to $\text{ PRVD}\!(\mathcal{P})$.

#### Corollary 20

$\text{ PRVD}\!(\mathcal{P})$ has a tree structure and has *Θ (n)* complexity.

#### Proof

Given a polygon edge in $\text{ PRVD}\!(\mathcal{P})$, its corresponding ray *r* trivially lies on the boundary of *vreg(r)* in $\text{ PRVD}\!(\mathcal{P})$. When truncating the diagram $\text{ DD }(\mathcal{S} _{\mathcal{P}})$ along the polygon boundary we derive $\text{ PRVD}\!(\mathcal{P})$, as proved in Lemma 18. By Lemma 19 each region in the diagram $\text{ DD }(\mathcal{S} _{\mathcal{P}})$ is connected, so the truncation trims each region at one occurrence only, i.e., the corresponding polygon edge. Hence, each region in $\text{ PRVD}\!(\mathcal{P})$ remains connected and appears along the polygon boundary only once, concluding the following. *□ *

We first show the following *monotonicity property* of the Voronoi regions.

#### Lemma 21

In $\text{ PRVD}\!(\mathcal{P})$, the Voronoi region of a site $r_i$ has the following property: the distance function on the boundary of the region is monotonically increasing from $r_i$, and from $r_{i+1}$, towards one point $r_i^*$ where it attains its maximum.

#### Proof

Recall that in Corollary 20 we showed that the region of $r_i$ consists of a single face incident to the polygon edge $p(r_i)p(r_{i+1})$.

**Fig. 19:**
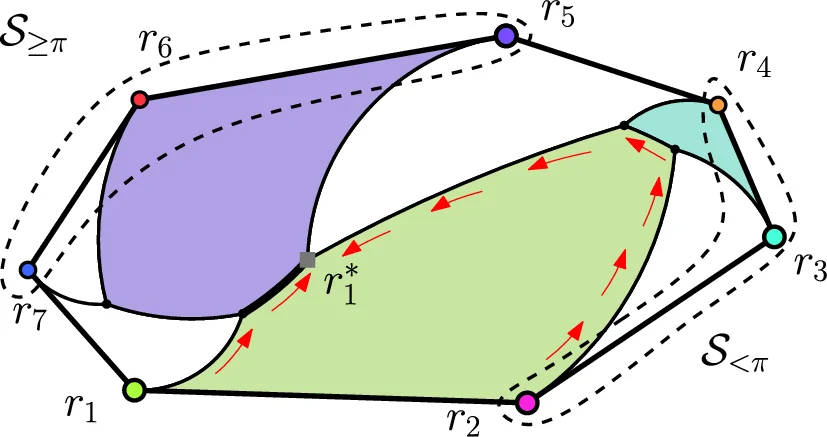
Illustration of the properties of a region $vreg(r_1)$. The distance along the boundary $\partial vreg(r_1)$ is increasing towards the maximum $r_1^*$. The sites in $\mathcal{S} _\mathcal{P} {\setminus} r_1$ are split in two sets $\{r_2,r_3,r_4\}$ and $\{r_5,r_6,r_7\}$. The regions of $r_1$ and $r_5$ split $\mathcal{P} {\setminus} \{vreg(r_5)\cup vreg(r_1)\}$ in two connected components

Consider the sequence of sites whose faces are adjacent to the face of $r_i$ in counterclockwise order. We show that: (i) this sequence is a subsequence of $(r_{i+1}, r_{i+2},..., r_n, r_1,...,r_{i-1})$, and (ii) the distance along the sequence is monotonically increasing towards a point realizing the maximum, denoted by $r_i^*$. Refer to Fig. 19 (where $r_i = r_1$).

(i). Let $r_j$ be a ray such that $vreg(r_i)$ is adjacent to $vreg(r_j)$. By Corollary 20, each region is connected and incident to its corresponding ray, so the union of $vreg(r_i)$ and $vreg(r_j)$ *splits* the convex polygon into two simply connected components (see $vreg(r_1) \cup vreg(r_5)$ in Fig. 19). Given a second ray $r_k$ whose region is adjacent to the region $vreg(r_i)$, it follows that both $r_k$ and the edge separating $vreg(r_i)$ and $vreg(r_k)$ have to be in the same connected component, as also $vreg(r_k)$ is connected (see $vreg(r_3)$ in Fig. 19). Thus, the order of $r_j$ and $r_k$ along the boundary of the polygon and the face of $r_i$ is the same.

(ii). We partition the set $\mathcal{S} _\mathcal{P} {\setminus} p_i$ in two depending on the angular difference with $p_i$; the set $\mathcal{S} _{\ge \pi }$ of rays with angular difference $\text{ diff}_{\angle }(r_j,r_i) \ge \pi$, and the set $\mathcal{S} _{<\pi }$ of rays with angular difference $\text{ diff}_{\angle }(r_j,r_i) < \pi$ (see the dashed curves in Fig. 19). Along the chain of rays $\mathcal{S} _{<\pi }$ (resp. $\mathcal{S} _{\ge \pi }$) the distance is increasing from $r_{i+1}$ (resp. $r_{i}$) towards the point realizing $r_i^*$.

We give an inductive argument for the monotonicity property along chain $\mathcal{S} _{<\pi }$, starting initially only with the Voronoi diagram of the two rays $r_i$ and $r_{i+1}$, and then incrementally adding more rays of the set $\mathcal{S} _{<\pi }$ in counterclockwise order. The base case follows directly from the properties of the bisectors, see Remark 5. Suppose we are now adding site $r_k$ to $\text{ RVD}\!(\{r_i,\dots ,r_{j-1}\})$. Because of property (i), if there is an edge between $r_i$ and $r_j$, then it is incident to $p(r_i)$, i.e., it is the last one along the chain of edges bounding face $vreg(r_i)$. Let *v* be the other endpoint of the edge between $r_i$ and $r_j$. Since $r_j \in \mathcal{S} _{<\pi }$, the distance along the edge $r_i$ and $r_j$ is monotonically increasing, from *v* to $p(r_i)$. Further, by the induction hypothesis, the distance along the chain of edges between $r_i$ and all sites bounding $vreg(r_i)$ in $\text{ RVD}\!(\{r_i,\dots ,r_{j-1}\})$ is monotonically increasing in counterclockwise order, from $r_{i+1}$ to *v*. The proof for $\mathcal{S} _{\ge \pi }$ is analogous, only the distance increases in clockwise order. *□ *

We prepare the discussion in Sect. 4.2 by giving the next three statements. The first one directly follows from Corollary 20 and Lemma21.

#### Corollary 22

$\text{ PRVD}\!(\mathcal{P})$ can be seen as a rooted tree, along which the distance of a point from its nearest ray is monotonically increasing, starting as 0 at the leaves (apices of the polygon) and attaining a maximum value at the root.

#### Corollary 23

For any vertex $u \in \text{ PRVD}\!(\mathcal{P})$, at least two incident edges have a distance increasing towards *u*.

#### Proof

Suppose, for the sake of contradiction, that there is a vertex *u* with two incident edges having distance decreasing towards *u*. These two edges are part of a chain of edges bounding a region $vreg(r_i)$. But this contradicts Lemma 21. *□ *

#### Lemma 24

Given an angle *c*, the set of all points in the interior of $\mathcal{P}$, which have distance at least *c* from their nearest site, forms a convex polygon *B*.

#### Proof

Observe that the set of all points at distance *c* from a ray of $\mathcal{S} _\mathcal{P}$ is a half-line. Thus, *B* is a convex polygon, since it is the intersection of the halfplanes defined by all the rays in $\mathcal{S} _\mathcal{P}$, after being rotated by *c*. *□ *

### A Simple *O(n łog n)*-Time Algorithm

We first describe a simple *O(nłog n)*-time algorithm to construct $\text{ PRVD}\!(\mathcal{P})$ employing a so-called *“collapse”* strategy: starting at the boundary of $\mathcal{P}$ (where all the points have distance zero to their nearest site), the algorithm gradually constructs the diagram adding edges and vertices in order of increasing distance until the vertex of maximum distance is reached. Examples of algorithms employing a similar collapse strategy include the farthest Voronoi diagrams of points [36], line segments [3], and convex sites in 3 dimensions [34]. All these collapse algorithms rely on a statement analogous to Corollary 22. We remark that the *O(nłog n)*-time algorithm of [2] finds the Brocard angle in a similar manner, without constructing $\text{ PRVD}\!(\mathcal{P})$.

**Fig. 20:**
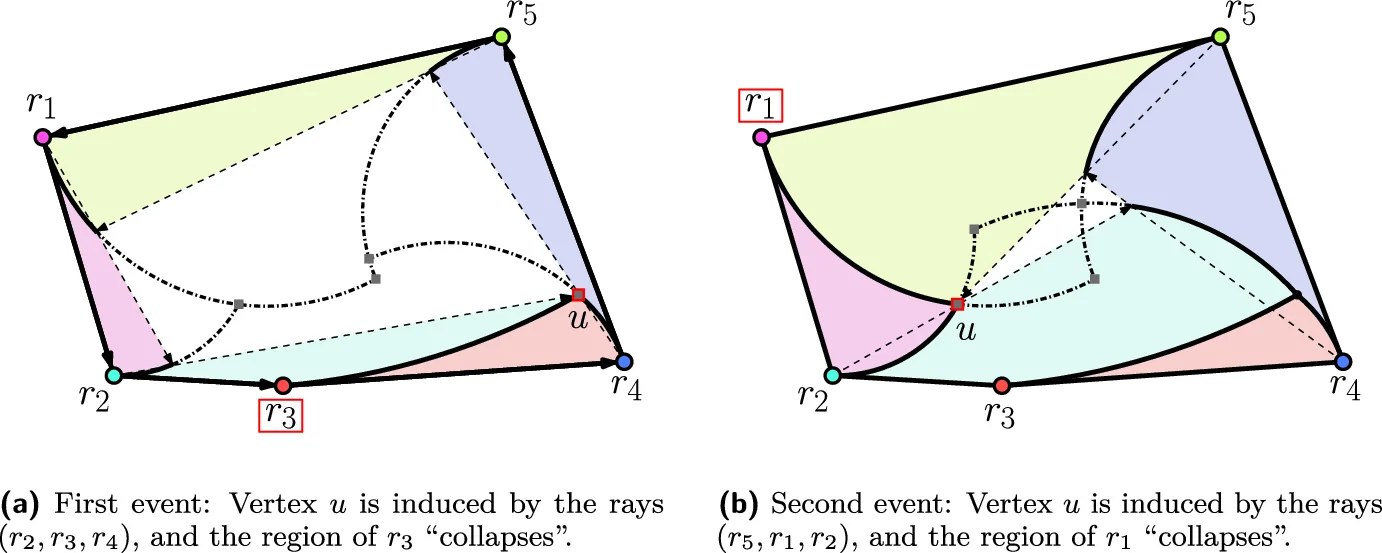
An illustration of the first two events of the *O(nłog n)*-time algorithm. At each event all the candidate vertices are illustrated; In the third event (not illustrated) there is only one candidate vertex induced by the rays $r_2,r_4,r_5$

We give a high level description of the collapse algorithm; refer to Fig. 20. The algorithm starts at the vertices of $\mathcal{P}$, which are all starting points of edges of $\text{ PRVD}\!(\mathcal{P})$, and creates a circular list of their incident edges. For every pair of edges that are consecutive in the circular order, their next intersection point is computed (the intersection of two circular arcs within the polygon), if one exists. Out of these intersection points, the one with minimum distance to its nearest site is the next vertex of the diagram.

This *collapse* event is processed by constructing the vertex and the edges leading to this vertex, then removing the edges from further consideration and starting a new edge. At the constructed vertex a face *collapses*, since it is fully constructed and will not be considered again. The new edge is part of the bisector of the two faces neighboring the collapsed face. This procedure, of computing and processing new *collapse* events, is repeated until all the remaining edges intersect in a single point. This last point is the vertex of $\text{ PRVD}\!(\mathcal{P})$ with maximum distance.

**Correctness of the algorithm**. The algorithm constructs the features of the diagram (vertices and edges) in increasing distance to their nearest site. First note that because of Lemma 24, the set of points with same distance to their closest site form a single cycle (see the dashed polygons in Fig. 20). So to keep track of the edges which are *“active” candidates* to be added to the diagram it suffices to use a circular list.

We argue that the algorithm correctly finds the next vertex, assuming that all vertices with smaller distance have already been constructed. By Lemma 23, every vertex has at least two edges with increasing distance towards it, thus, the next vertex is the intersection of a pair of edges currently in the circular list. Such a pair of edges needs to be consecutive, as otherwise, the cyclicity of Lemma 24 would be violated. Since we are constructing the diagram in increasing distance, the algorithm picks the candidate vertex with smallest distance.

Finally, we argue that the algorithm does not miss any candidate edge. This is because the algorithm considers a new candidate edge at each vertex event, and edges can only start at vertices, since the distance function does not exhibit local minima along an edge; see the monotonicity property of Remark 5.

#### Proposition 25

The “collapse” algorithm constructs $\text{ PRVD}\!(\mathcal{P})$ in *O(nłog n)* time.

#### Proof

The time complexity analysis is straightforward. The algorithm takes *O(nłog n)* time to sort the first *n* events. Then, there are *n-2* events and each event takes *O(łog n)* time, if we use a min priority queue. Thus, the algorithm takes *O(nłog n)* time overall. *□ *

### An Optimal *Θ (n)*-Time Algorithm

We now show that $\text{ PRVD}\!(\mathcal{P})$ can be constructed in optimal *Θ (n)*-time. We have already shown that $\text{ PRVD}\!(\mathcal{P})$ has a tree structure and connected Voronoi regions. Despite its simple structure, however, $\text{ PRVD}\!(\mathcal{P})$ does not fall under the framework of *abstract Voronoi diagrams* [24], therefore, we cannot directly use the available machinery under this framework. Instead, we split the problem into sub-problems, where each sub-problem falls under the abstract Voronoi diagram framework, construct diagrams for these subproblems, and perform a final (non-standard) merge.

**Fig. 21:**
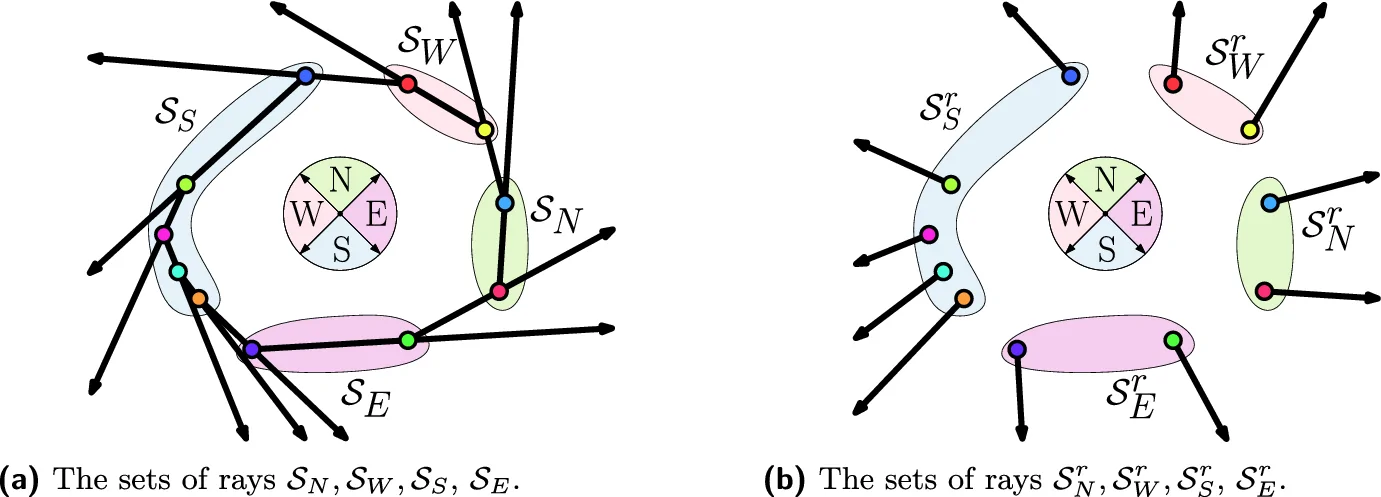
The partitioning of the set of rays in $\mathcal{S} _{\mathcal{P}}$ before and after rotation

Our algorithm can be briefly described as follows, see also the pseudocode in Algorithm 1. In the first step, we partition $\mathcal{S} _{\mathcal{P}}$ into four sets $\mathcal{S} _{N}, \mathcal{S} _{W}, \mathcal{S} _{S}$ and $\mathcal{S} _{E}$ of consecutive rays, depending on whether a ray faces *north*, *west*, *south* or *east* respectively; see an example in Fig. 21a.

**Fig. 22:**
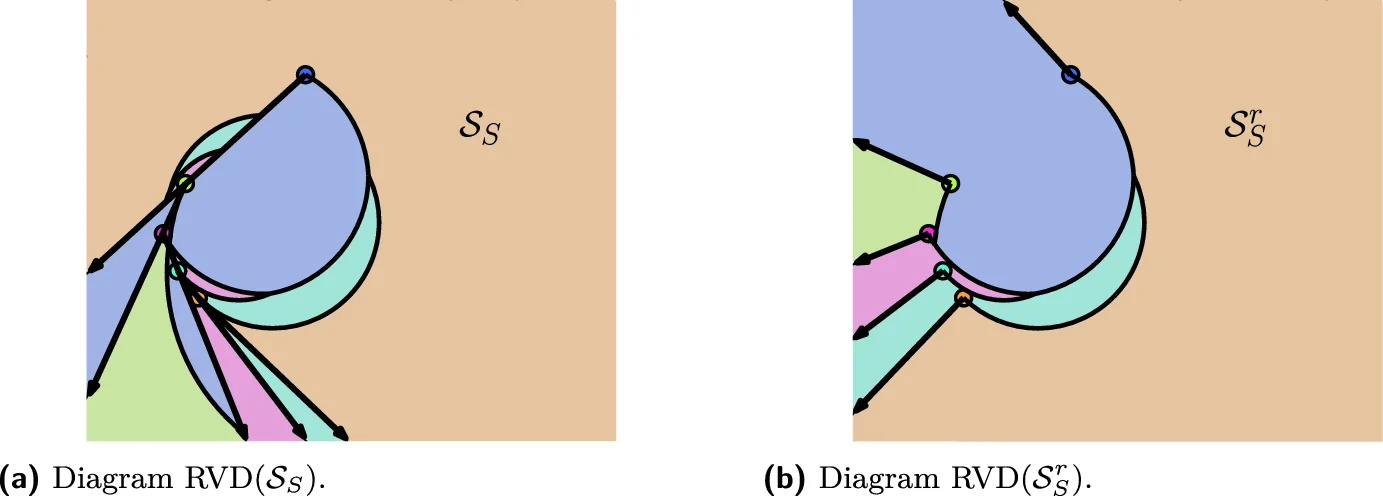
Voronoi diagrams of a set $\mathcal{S} _S$ and the set $\mathcal{S} _S^r$ (after a clockwise rotation by *π /2*)

In the second step, we transform each set $\mathcal{S} _d$, $d \in \{N,W,S,E \}$ into a set $\mathcal{S} ^r_d$, where each ray in $\mathcal{S} _d$ is rotated clockwise by an angle of *π /2*; see Fig. 21b. We then construct each diagram $\text{ RVD}\!(\mathcal{S} ^r_d)$ independently as a special instance of abstract Voronoi diagrams; see Fig. 22. Finally, we merge the four diagrams and obtain $\text{ PRVD}\!(\mathcal{P})$. The merging is done in two phases; see Figs. 25 and 26.

**Algorithm 1:**
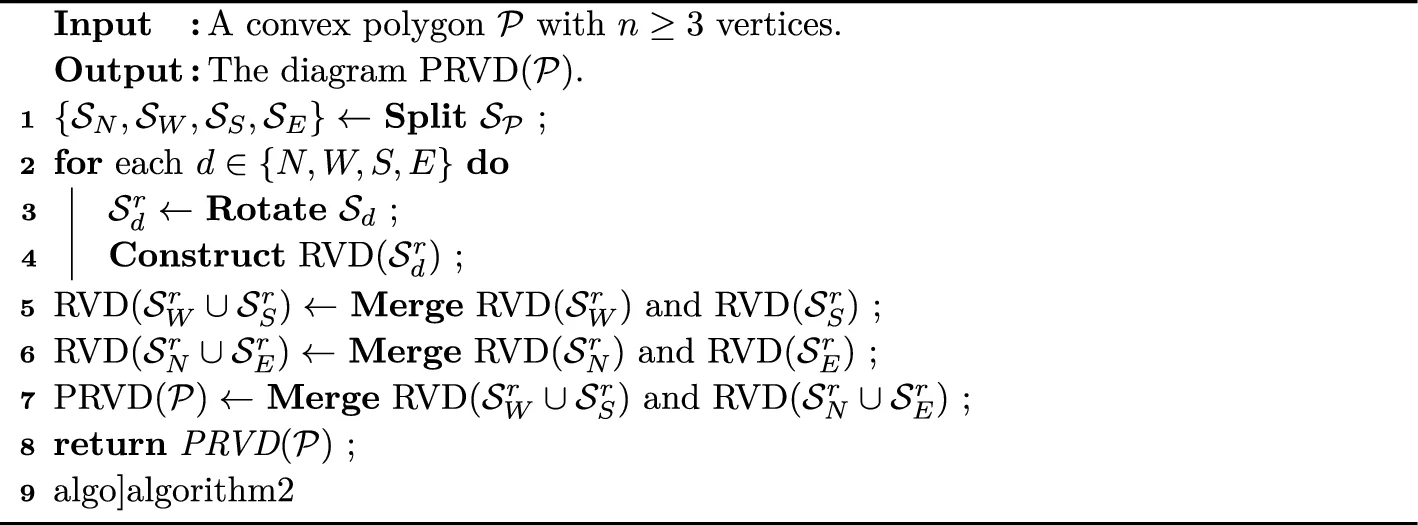
*Θ (n)*-time algorithm to construct $\text{ PRVD}\!(\mathcal{P})$.

We describe in detail the construction of the four diagrams in Sect. 4.4 and the merging phase in Sect. 4.5. Consequently we derive the following theorem.

#### Theorem 26

Given a convex polygon $\mathcal{P}$, we can construct $\text{ PRVD}\!(\mathcal{P})$ in deterministic optimal *Θ (n)* time.

### *Θ (n)*-Time Algorithm: Constructing the Four Diagrams

We use the framework of *abstract Voronoi diagrams* [24, 25]. To comply with this framework, a system of angular bisectors must satisfy the following three *axioms*:

- **(A1)** The bisector $b_{\angle }(r,s)$, $\forall r,s \in \mathcal{S}$, is an unbounded simple curve, homeomorphic to a line.
- **(A2)** The region *vreg(r)* in $\text{ RVD}\!(\mathcal{S} ')$, $\forall \mathcal{S} ' \subseteq \mathcal{S}$ and $\forall r \in \mathcal{S} '$, is connected.
- **(A3)** The union of the closures of all regions in $\text{ RVD}\!(\mathcal{S} ')$, $\forall \mathcal{S} ' \subseteq \mathcal{S}$, covers $\mathbb {R}^2$.

Observe that a subset $\mathcal{S} _d$ (and hence the set $\mathcal{S} _{\mathcal{P}}$) need not satisfy axiom (A2); see the disconnected Voronoi regions in Fig. 22a.

Consider each transformed subset of rays $\mathcal{S} ^r_d$, $d \in \{N,W,S,E \}$, where each ray is rotated clockwise by an angle of *π /2*. We will show that each set $\mathcal{S} ^r_d$ satisfies the aforementioned axioms. The intuition behind the clockwise rotation comes from the fact that only the circular parts of bisectors appear in $\text{ PRVD}\!(\mathcal{P})$, which remain the same under a uniform rotation. An example of a diagram that satisfies axiom (A2) after a clockwise *(π /2)*-rotation is shown in Fig. 22b.

**Fig. 23:**
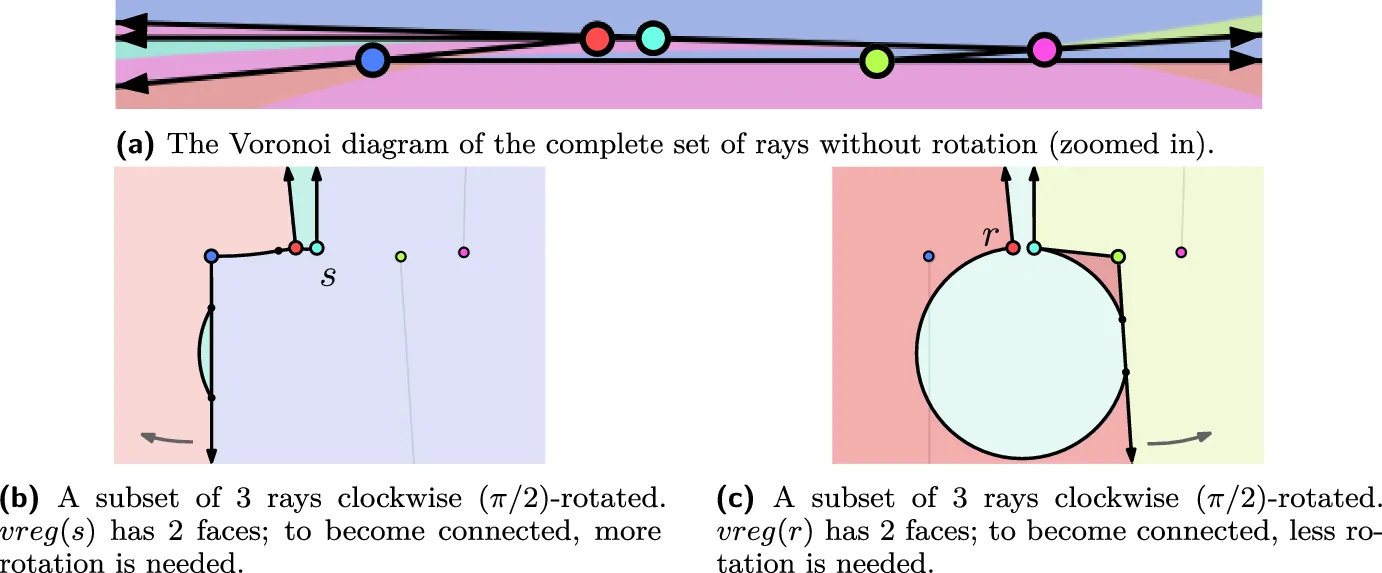
An example of a (thin) polygon with 5 vertices which justifies Remark 27

A clockwise *(π /2)*-rotation by itself is not always sufficient for the entire set $\mathcal{S} _{\mathcal{P}}$ to satisfy axiom (A2), and this is justified by the example in Fig. 23: given the set $\mathcal{S} _\mathcal{P}$ of 5 rays in Fig. 23a, there exists a subset of 3 rays which needs more rotation in order to have all regions connected, see *vreg(s)* in Fig. 23b, and a subset of 3 rays which needs less rotation, see *vreg(r)* in Fig. 23c; hence, the reason to split $\mathcal{S} _\mathcal{P}$ appropriately.

#### Remark 27

There are sets of rays $\mathcal{S} _{\mathcal{P}}$ for which there exists no unique angle to rotate the rays so that axiom (A2) is satisfied.

Note that we partitioned $\mathcal{S} _\mathcal{P}$ in a way such that any two rays *r* and *s* in a set $\mathcal{S} ^r_d$ have an angular difference of at most *π /2*, i.e., $\min \{ \text{ diff}_{\angle }(r,s), \text{ diff}_{\angle }(s,r) \} \le \pi /2$. This is a key property in proving the following lemma.

#### Lemma 28

The system of bisectors of $\mathcal{S} ^r_d$ satisfies the axioms (A1)-(A3).

#### Proof

We prove each of the three axioms separately.

**(A1):** Let *r*, *s* be a pair of rays in $\mathcal{S} ^r_d$. We show that *r* and *s* do not intersect. Then, by Lemma 3, the bisector of two non-intersecting rays is an unbounded simple curve.

**Fig. 24:**
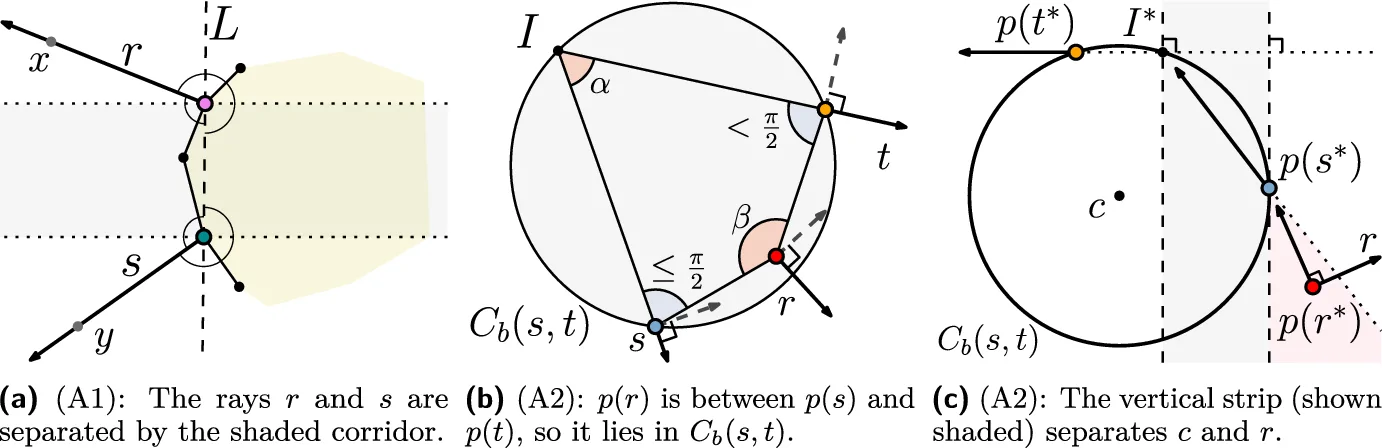
Illustrations for the proof of Lemma 28

Let $x \in r {\setminus} \{p(r)\}$ (resp. $y \in s {\setminus} \{p(s)\}$), denote a point lying on *r* (resp. *s*), and let *L* be the line passing through *p(r)* and *p(s)*; see Fig. 24a. Due to the convexity of the polygon, it follows that *∠ (x,p(r),p(s))* and *∠ (p(s),p(r),x)* are greater than or equal to *π /2*; hence *r* lies in the closed halfplane orthogonal to *L* incident to *p(r)* which does not contain *p(s)*. Analogously, *s* lies in the closed halfplane orthogonal to *L* incident to *p(s)* that does not contain *p(r)*. Thus, the horizontal strip defined by the two halfplanes (shown shaded in Fig. 24a) separates *r* and *s*, and so, they do not intersect.

**(A2):** It suffices to prove the property for any subset of $\mathcal{S} ^r_d$ of size three [25]. By Lemma 8, each Voronoi region has exactly one unbounded face, so if a region is disconnected, then it must have at least one bounded face. The diagram of three rays can have at most one proper Voronoi vertex, as shown in Lemma 7. Thus, a bounded face in the diagram cannot be bounded by only the edges incident to that single proper vertex, and so, such a bounded face must be incident to a ray. Hence, it suffices to show that none of the three rays can intersect twice the bisecting circle of the other two.

Let $\{ r,s,t \}$ be a subset of 3 rays of $\mathcal{S} ^r_d$. We will show that no ray in $\{r,s,t\}$ intersects twice the bisecting circle defined by the other two rays in $\{r,s,t\}$. We prove this for *r*, i.e., *r* does not intersect twice $C_{b}(s,t)$; the cases of *s*, *t* are analogous. Without loss of generality we assume that $\text{ diff}_{\angle }(t,s)<\text{ diff}_{\angle }(s,t)$. We divide the proof into three cases, depending on the relative position of *p(r)*.

1. If *p(r)* lies in the interior of $C_{b}(s,t)$, then *r* intersects $C_{b}(s,t)$ exactly once and the claim follows.
2. Assume that *p(r)* appears between *p(s)* and *p(t)* along the convex polygon chain, and let *I* be the intersection of the supporting lines *l*(*s*) and *l*(*t*); see Fig. 24b. Because of the rotation of rays, we have $\angle (p(r),p(s),I)\le \pi /2$ and $\angle (I,p(t),p(r))<\pi /2$. Thus, by the properties of cyclic quadrilaterals^3^, *p(r)* lies in the interior of $C_{b}(s,t)$, the circle through *p(s)*, *p(t)* and *I*, see that $\alpha +\beta >\pi$ in Fig. 24b). Thus, *r* intersects $C_{b}(s,t)$ once.
3. It remains to study the case where *p(r)* lies outside $C_{b}(s,t)$, and *p(r)* appears before or after both *p(s)* and *p(t)*, in counterclockwise order. We consider the case when *p(r)* appears before *p(s)* and *p(t)*. The other case is analogous.
  Let *c* denote the center of $C_{b}(s,t)$, let $r^*$ (resp. $s^*$, $t^*$) denote the ray *r* (resp. *s*, *t*) rotated counterclockwise by *π /2* around its apex, and let $I^*$ denote the intersection point between the supporting lines $l(s^*)$ and $l(t^*)$. Without loss of generality, we assume that $t^*$ is a horizontal ray pointing left; refer to Fig. 24c. If *r* intersects twice $C_{b}(s,t)$, then *c* lies to the right of the (directed) line $l(r^*)$, so it suffices to prove that *c* lies to the left of $l(r^*)$. To show this, observe that $p(r^*)$ lies to the right of the vertical line through $p(s^*)$ (since $\text{ diff}_{\angle }(t^*,r^*) \le \pi /2$), and to the left of $l(s^*)$ (since $r^*$ and $s^*$ are induced by a convex polygon); see the red region in Fig. 24c. On the other hand, the vertical line through $I^*$ separates *C* from $p(r^*)$. Hence, *c* lies to the left of $r^*$, proving the claim.

**(A3):** The diagram $\text{ RVD}\!(\mathcal{S} ^r_d)$ is defined by distance functions, one for each site in $\mathcal{S} ^r_d$, whose domain is the entire plane. Hence, any point in the plane must belong to the closure of some region of $\text{ RVD}\!(\mathcal{S} ^r_d)$. *□ *

Since each Voronoi region is connected and unbounded, and since $\text{ RVD}\!(\mathcal{S})$ is connected (Lemma 9), we can infer the following.

#### Corollary 29

$\text{ RVD}\!(\mathcal{S} ^r_d)$ is a tree of $\Theta (\vert \mathcal{S} ^r_d\vert )$ complexity.

We can obtain an *O*(*n*)-time algorithm to construct $\text{ RVD}\!(\mathcal{S} ^r_d)$ by showing that the bisector system of $\mathcal{S} ^r_d$ satisfies the more restricted *Hamiltonian abstract Voronoi diagram* framework [26]. To this goal, in addition to satisfying axioms (A1)-(A3), the following axiom must also be satisfied [26]:

- **(A4)** There exists a simple curve $\mathcal{H}$ of constant complexity such that $\mathcal{H}$ visits each region *vreg(r)* in $\text{ RVD}\!(\mathcal{S} ')$, $\forall \mathcal{S} ' \subseteq \mathcal{S}$ and $\forall r \in \mathcal{S} '$, exactly once. $\mathcal{H}$ can be closed or unbounded.

Hence for our problem, it suffices to determine a curve $\mathcal{H}$ satisfying (A4).

#### Lemma 30

$\text{ RVD}\!(\mathcal{S} ^r_d)$ can be constructed in deterministic $\Theta (\vert \mathcal{S} _d\vert )$ time.

#### Proof

We show that $\mathcal{S} ^r_d$ satisfies axiom (A4) as defined above; the linear time algorithm is then a direct corollary of the existing results [26].

Let $\mathcal{H}$ be a circle of sufficient large radius, such that all bisecting circles lie entirely in the interior of $\mathcal{H}$. For any $\mathcal{S} ' \subseteq \mathcal{S} ^r_d$, the diagram $\text{ RVD}\!(\mathcal{S} ')$ is a tree (Corollary 29), so its faces are all unbounded. By its definition, $\mathcal{H}$ does not intersect any bisecting circle, hence, $\mathcal{H}$ visits each region of $\text{ RVD}\!(\mathcal{S} ')$ exactly once, and more specifically, a change in the visited region takes place when $\mathcal{H}$ intersects a ray.

The ordering of the unbounded faces of $\text{ RVD}\!(\mathcal{S} ^r_d)$ follows the ordering of the respective vertices along the polygon $\mathcal{P}$, and this is maintained for any $\mathcal{S} ' \subset \mathcal{S} ^r_d$. In addition, the ordering of the vertices of $\mathcal{P}$ is part of the input, concluding the proof. *□ *

### *Θ (n)*-Time Algorithm: Merging the Four Diagrams

We now merge all four diagrams to obtain $\text{ PRVD}\!(\mathcal{P})$. Note that the merging process requires attention because that any diagram consequent merged diagram, other than the initial ones, does not fall under the framework of abstract Voronoi diagrams.

**Fig. 25:**
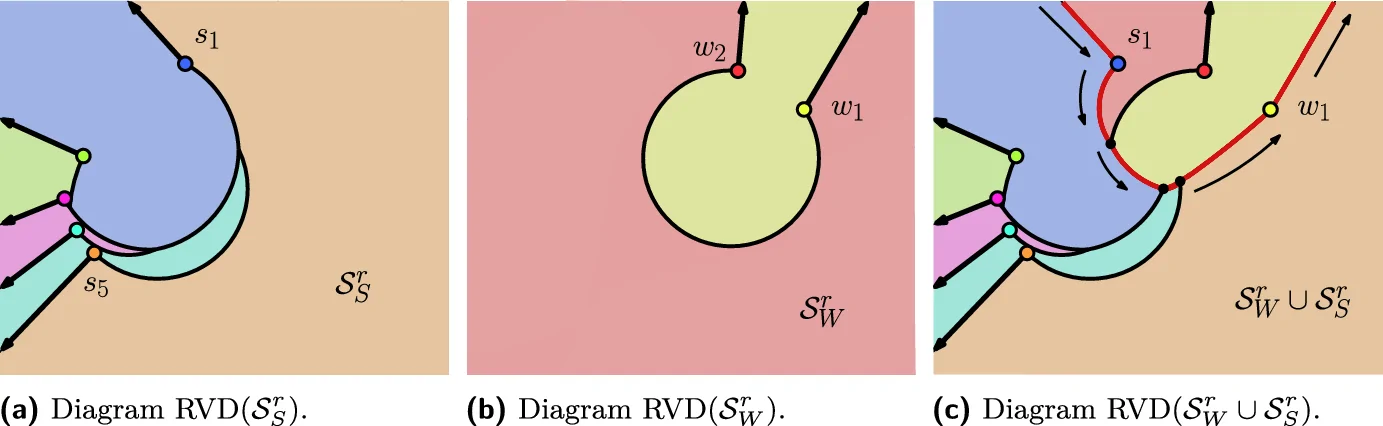
First merging phase: merging $\text{ RVD}\!(\mathcal{S} ^r_{W})$ and $\text{ RVD}\!(\mathcal{S} ^r_{S})$. The red edges in **c** highlight the merge curve, and the arrows schematize tracing (starting along $s_1$ at infinity and ending at $w_1$)

**Fig. 26:**
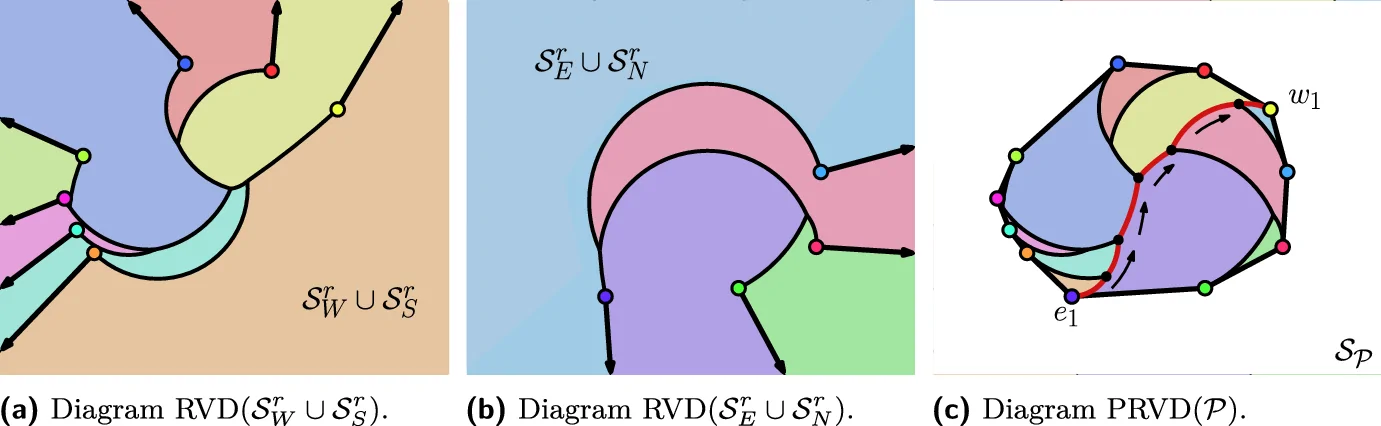
Second merging phase: merging $\text{ RVD}\!(\mathcal{S} ^r_{W} \cup \mathcal{S} ^r_{S})$ and $\text{ RVD}\!(\mathcal{S} ^r_{E} \cup \mathcal{S} ^r_{N})$ restricted to $\mathcal{P}$. The red edges in **c** highlight the merge curve, and the arrows schematize tracing (starting at $p(e_1)$ and ending at $p(w_1)$)

Our merging process consists of two phases. In the *first phase* we merge $\text{ RVD}\!(\mathcal{S} ^r_{W})$ and $\text{ RVD}\!(\mathcal{S} ^r_{S})$ to obtain $\text{ RVD}\!(\mathcal{S} ^r_{W} \cup \mathcal{S} ^r_{S})$; and respectively for $\text{ RVD}\!(\mathcal{S} ^r_{E} \cup \mathcal{S} ^r_{N})$; see Fig. 25. In the *second phase* we merge $\text{ RVD}\!(\mathcal{S} ^r_{W} \cup \mathcal{S} ^r_{S})$ and $\text{ RVD}\!(\mathcal{S} ^r_{E} \cup \mathcal{S} ^r_{N})$, however, restricted in the interior of $\mathcal{P}$, to obtain $\text{ PRVD}\!(\mathcal{P})$; see Fig. 26. We first give a high level outline of the merging process. Then, we proceed with a detailed description of the different procedures of the algorithm, with their proofs, and we conclude the time complexity analysis. We will ultimately prove the following.

#### Lemma 31

Given $\text{ RVD}\!(\mathcal{S} _d)$ for all $d \in \{N,W,S,E \}$, we can merge the four diagrams to obtain $\text{ PRVD}\!(\mathcal{P})$ in *Θ (n)* time.

**Outline of the merging process**. We describe how to merge $\text{ RVD}\!(\mathcal{S} ^r_{W})$ with $\text{ RVD}\!(\mathcal{S} ^r_{S})$; merging $\text{ RVD}\!(\mathcal{S} ^r_{E})$ with $\text{ RVD}\!(\mathcal{S} ^r_{N})$ is analogous. Let $w_1,\dots ,w_k$ be the rays in $\mathcal{S} ^r_W$ and let $s_1,\dots ,s_l$ be the rays in $\mathcal{S} ^r_S$ as they appear in counterclockwise order along the boundary of $\mathcal{P}$. The rays in $\mathcal{S} ^r_E$ and $\mathcal{S} ^r_N$ in the same order are denoted $e_1,\dots ,e_p$ and $n_1,\dots ,n_q$, respectively.

We need to construct the *merge curve* of the two diagrams, which partitions $\mathbb {R}^2$ in two parts, and keep from one side the diagram $\text{ RVD}\!(\mathcal{S} ^r_{W})$ and from the other $\text{ RVD}\!(\mathcal{S} ^r_{S})$; refer to Fig. 25c, where the red edges illustrate the merge curve. In the first phase, the merge curve consists of the two rays $s_1$ and $w_1$, and the set of circular edges of $\text{ RVD}\!(\mathcal{S} ^r_{W} \cup \mathcal{S} ^r_{S})$ equidistant to sites $w \in \mathcal{S} ^r_W$ and $s \in \mathcal{S} ^r_S$. The set of circular edges forms a single connected chain bounded by $p(s_1)$ and $w_1$ (as it will be shown in the sequel). We denote the set of circular edges in a merge curve by $E_C$.

In the second phase, we perform merging inside the polygon $\mathcal{P}$, merging $\text{ RVD}\!(\mathcal{S} ^r_{W} \cup \mathcal{S} ^r_{S})\cap \mathcal{P}$ and $\text{ RVD}\!(\mathcal{S} ^r_{E} \cup \mathcal{S} ^r_{N})\cap \mathcal{P}$. Since the computation is restricted in $\mathcal{P}$, the merge curve consists of only the circular edges in $E_C$, which is a single connected chain bounded by $p(e_1)$ and $p(w_1)$; see Fig. 26c.

Following, we describe in detail the merging process and prove the correctness of our statements. Constructing the merge curve is based on finding a starting point along the merge curve, and then *tracing* it, as typically done in standard Voronoi diagrams (see, e.g., [4]).

**Tracing along the rays (first merging phase)**. As already mentioned, the merge curve at the first merging phase consists of the two rays $s_1$ and $w_1$ and the set of circular edges $E_C$. We start tracing the merge curve, by a point on the ray $s_1$ at infinity. Tracing along $s_1$ can be done trivially, this is because the ray lies entirely in $vreg(w_k)$. To see that, consider the set $\mathcal{S} _W$ (before rotation) and continuously clockwise rotate all rays by an angle of *π /2*. During this process, $w_k$ does not intersect any of the rays in $\mathcal{S} _W$, hence $s_1 \in vreg(w_k)$.

**Fig. 27:**
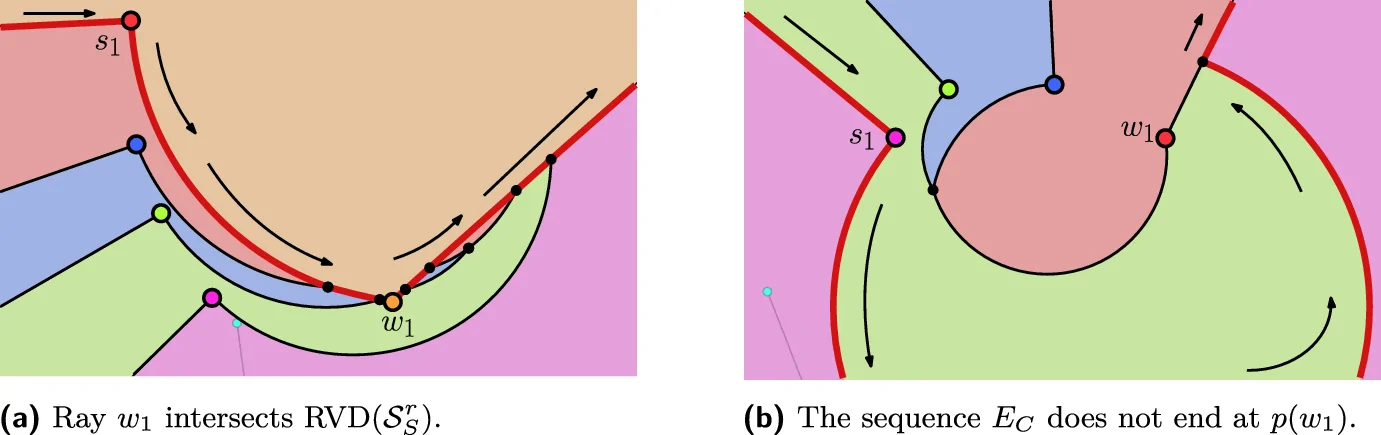
Two special cases of merging two diagrams $\text{ RVD}\!(\mathcal{S} ^r_{W})$ and $\text{ RVD}\!(\mathcal{S} ^r_{S})$

After ray $s_1$, tracing continues along the circular edges $E_C$ (described in the next paragraph) and finally it reaches ray $w_1$. Tracing along the ray $w_1$ is done in a different way. In contrast to $s_1$, the ray $w_1$ may intersect many circular edges of $\text{ RVD}\!(\mathcal{S} ^r_S$), each inducing a vertex on $w_1$; see e.g., Fig. 27a. To identify such vertices, we intersect $w_1$ with $\text{ RVD}\!(\mathcal{S} ^r_S)$. This can be easily done in $O(|\mathcal{S} _S|)$ time, as $\text{ RVD}\!(\mathcal{S} ^r_S)$ is proved to be a tree (see Corollary 29). Further, the curve $E_C$ might intersect $w_1$ at some point other than $p(w_1)$; see e.g., Fig. 27b. In this case the aforementioned search should start from that point.

**Tracing the sequence of circular edges** $E_C$ **(both first and second merging phases)**. We now describe how to trace $E_C$ in $\Theta (|E_C|)$ time by adapting the standard procedure for tracing a merge curve in Voronoi diagrams, as done for example for bisectors of points [4], to angular bisectors. To establish correctness, however, we still need to prove that no *backtracking* needs ever be done during merging, i.e., while tracing any portion of a Voronoi region is scanned at most once; we prove this later, in Lemma 32.

**Fig. 28:**
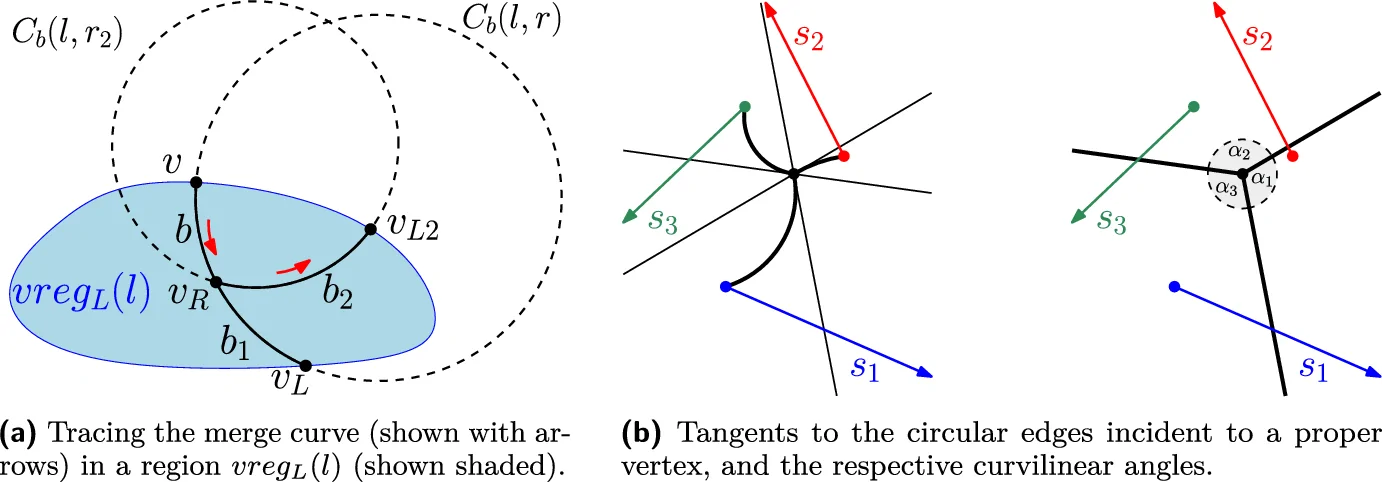
Illustration of the tracing process while merging $E_C$, and the non-necessity to backtrack

Suppose we are in the process of merging two ray Voronoi diagrams, tracing a merge curve, whose main portion of circular edges is denoted by $E_C$. Let *L* be the set of rays defining the diagram to the left of the curve $E_C$ and *R* the set to the right hand side. Without loss of generality assume that we are tracing $E_C$ from top to bottom; refer to Fig. 28a. Let $vreg_L(l)$ denote the Voronoi region of site *l ∈ L* within $\text{ RVD}\!(L)$; respectively for $vreg_R(r)$, *r∈ R*. Suppose that the current edge *b* of $E_C$ has just entered a region $vreg_L(l)$ at point *v*. Let *r* be the site of the right diagram such that the edge *b* lies in the region $vreg_R(r)$. We determine the points $v_L$ (resp. $v_R$) where *b* leaves the region $vreg_L(l)$ (resp. $vreg_R(r)$). The point $v_L$ is found by scanning the boundary of $vreg_L(l)$ clockwise starting from *v*. The point $v_R$ is found by scanning the boundary of $vreg_R(r)$ counterclockwise starting from *v*. Without loss of generality assume that vertex $v_R$ is reached first, which then describes the endpoint of edge *b*.

$E_C$ continues from $v_R$ with another edge $b_2$ along the bisector $b_{\angle }(l,r_2)$, where $r_2$ is another site in *R*. To determine $v_{L2}$, we scan the boundary of $vreg_L(l)$, starting from $v_L$ and moving clockwise. This is shown correct in the following lemma, which establishes that $v_{L2}$ cannot be on the boundary of $vreg_L(l)$ that has already been scanned. Analogously for $v_{R2}$.

#### Lemma 32

There is no need to backtrack while tracing the sequence of circular edges $E_C$.

#### Proof

Let *v*, $v_L$ and $v_{L2}$ be the points as defined in the above description of tracing $E_C$. To prove that there is no need for backtracking, it suffices to prove that the points *v*, $v_L$ and $v_{L2}$ appear in counterclockwise order along the boundary of face $vreg_L(l)$; see Fig. 28a.

The *curvilinear angle* between two intersecting curves is the angle between their two tangents at the point of intersection. Each proper vertex of the diagram, has degree 3, so, the three edges incident to a vertex induce three curvilinear angles; see Fig. 28b. Each such curvilinear angle can be seen as the angle at the intersection of two halfplanes. Hence, each such angle is less than *π *.

The point $v_R$ is a proper vertex in the merged diagram. Therefore, the curvilinear angle $\angle (v_{L2},v_R,v)$ between the edges $b_2$ and *b* is less than *π *. On the other hand, the angle $\angle (v_L,v_R,v)$ between the edges $b_1$ and $b_2$ is exactly *π * as both edges lie on the same bisector. Within the polygon, two related bisectors intersect at most once. Thus, the edge $b_2$ has to hit the boundary of $vreg_L(l)$ after $v_L$ but before *v* in counterclockwise order. *□ *

Since there is no backtracking required to trace $E_C$, the tracing takes $\Theta (|E_C|)$ time.

**Correctness of the construction of** $E_C$. Following, we show the correctness of some claims used earlier without proof. More specifically, we show that ${\textbf {(i)}}$ the chain $E_C$ is incident to $w_1$, and $p(s_1)$ in the first phase, and to $p(e_1)$ in the second phase; ${\textbf {(ii)}}$ the chain constructed is the complete curve $E_C$, i.e., there are no other connected components left to identify;

(i) In the first merging phase, considering tracing the chain $E_C$ starting at $p(s_1)$. The distance at $p(s_1)$, is exactly *π /2*, and it is monotonically increasing. Further, consider the polygonal chain $P^*$ consisting of the line segments $\overline{p(w_1)p(w_2)},$ $\overline{p(w_2)p(w_3)},\dots ,$ $\overline{p(w_k)p(s_1)},$ $\overline{p(s_1)p(s_2)}, \dots ,$ $\overline{p(s_{l-1})p(s_l)}$ and the ray $s_l$. The distance of any point on $\mathcal{P} ^*$ to its nearest ray is *π /2*. Hence, as the distance along the chain $E_C$ is increasing, the only possibility for this chain to end up is at $w_1$. Similarly, in the second merging phase, the distance of any point on the complete polygon $\mathcal{P}$ to its nearest ray is *π /2*, and hence $E_C$ is bounded by $p(e_1)$ and $p(w_1)$.
(ii) To prove this statement we use the disk diagram, defined in Sect. 4.1. By Lemma 8, each region has exactly one unbounded face, so if there exists another connected component in $E_C$, it has to be bounded. A second unbounded component would imply that a Voronoi region has two unbounded faces. Suppose that the merge curve has another bounded connected component. Such a component is bounded entirely by circular edges, and these edges are induced by the respective bisecting circles of the bisectors. Since the bisecting circles of the disk diagrams are supersets of the circular edges appearing in $\text{ RVD}\!(\mathcal{S} ^r_{W} \cup \mathcal{S} ^r_{S})$, $\text{ RVD}\!(\mathcal{S} ^r_{E} \cup \mathcal{S} ^r_{N})$ and $\text{ PRVD}\!(\mathcal{P})$, such a bounded component would also appear in the respective disk diagrams, a contradiction to Lemma 19.

Suppose now that the merge curve has a component which is bounded from one side by a ray. In the final merging step this is not possible, as on $\text{ PRVD}\!(\mathcal{P})$ all points on an edge/ray of $\mathcal{P}$ belong to the region of the respective ray. In the initial merging step (assuming that we merge $\text{ RVD}\!(\mathcal{S} ^r_{W})$ with $\text{ RVD}\!(\mathcal{S} ^r_{S})$), for every ray $s_i \in \mathcal{S} ^r_{S}$, except from $w_1$, the complete right side of the ray is incident to $vreg(s_{i-1})$. This can be proved with the same argument used to show that $s_1 \in vreg(w_k)$ (where $s_1$ is the first ray of $\mathcal{S} ^r_S$, and $w_k$ is the last ray of $\mathcal{S} ^r_W$, respectively). As a result, no bounded component of $E_C$ could be incident to a ray. Hence, $E_C$ is a single unbounded chain.

From the above discussion we can infer that the merge curve does not induce bounded faces in the resulting diagram, except from ray $w_1$ in the first merging phase. As a result in the first merged diagram $\text{ RVD}\!(\mathcal{S} ^r_{W} \cup \mathcal{S} ^r_{S})$, each ray $s_i \in \mathcal{S} ^r_S$ can have at most two faces (the unbounded one and the one incident to $w_1$) and $vreg(w_i)$ is connected for any $w_i \in \mathcal{S} ^r_W$. On the contrary in $\text{ PRVD}\!(\mathcal{P})$, $vreg(r_i)$ is connected for any $r_i \in \mathcal{S} _{\mathcal{P}}$.

**Overall time complexity**. In the first step, tracing the rays $s_1$ and $n_1$ takes *Θ (1)* time, and tracing the rays $w_1$ and $e_1$, takes $\Theta (|\mathcal{S} _{S}|)$ time and $\Theta (|\mathcal{S} _{N}|)$ time, respectively. Tracing the curve $E_C$ takes $O(|\mathcal{S} _{W}| + |\mathcal{S} _{S}|)$ time, and $O(|\mathcal{S} _{E}| + |\mathcal{S} _{N}|)$ time, respectively; so in total the first step requires *O*(*n*) time. The final step requires *O*(*n*) time to trace $E_C$ and *Θ (n)* to restrict the diagram into $\mathcal{P}$. So, the overall merging of the four diagrams takes *Θ (n)* time.

Putting everything together, we can trivially split $\mathcal{S} _{\mathcal{P}}$ into four sets in *Θ (n)* time, we can construct the four diagrams in *Θ (n)* time (Lemma 30), and we can merge them in *Θ (n)* time (Lemma 31). So, we can summarize (and re-state), Theorem 26, the main result of this section as follows.

#### Theorem 26

Given a convex polygon $\mathcal{P}$, we can construct $\text{ PRVD}\!(\mathcal{P})$ in deterministic optimal *Θ (n)* time.

### Brocard Illumination of a Convex Polygon

We now turn to the Brocard illumination problem of a convex polygon $\mathcal{P}$. Our goal is to find the Brocard angle of $\mathcal{P}$, which is

$$\begin{aligned} & \alpha ^*= \max _{x \in \mathcal{P}}\min _{r \in \mathcal{S} _\mathcal{P}}d_{\angle }(x,r). \end{aligned}$$

**Fig. 29:**
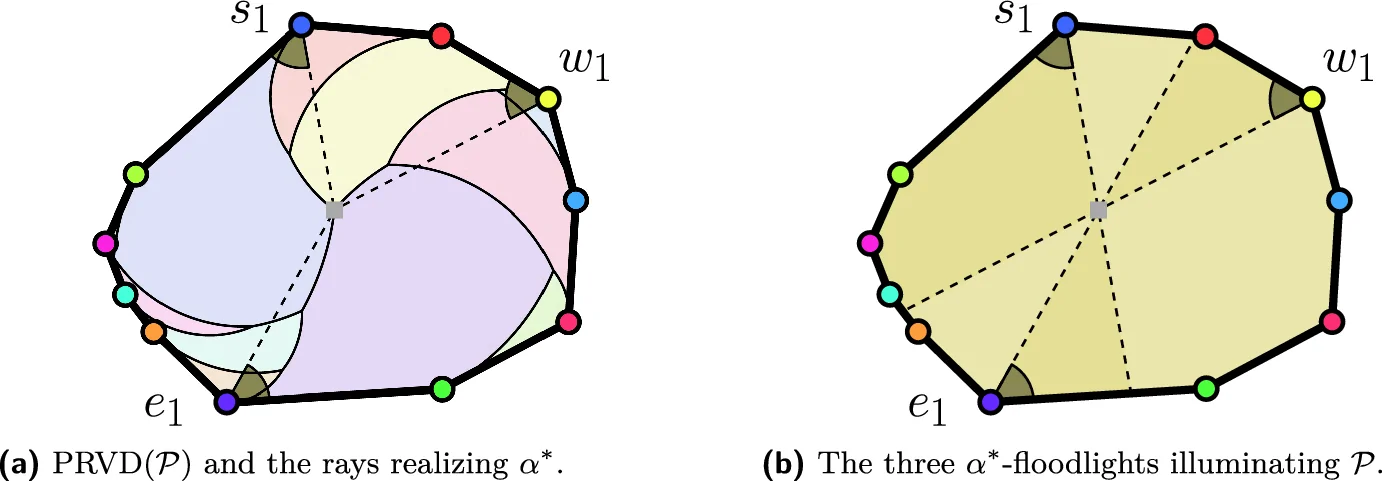
The Brocard angle $\alpha ^*$ of a polygon $\mathcal{P}$ realized by $(e_1,w_1,s_1)$

Observe that the diagram $\text{ PRVD}\!(\mathcal{P})$ is a subset of $\text{ RVD}\!(\mathcal{S} _\mathcal{P})$, hence Proposition 13 applies also in this setting, and so $\alpha ^*$ is realized on $\text{ PRVD}\!(\mathcal{P})$. However, since the diagram is strictly confined into $\mathcal{P}$, the point realizing the Brocard angle, can only lie on a vertex equidistant to 3 rays; see an example in Fig. 29a.

Similarly to the setting in $\mathbb {R}^2$, to find $\alpha ^*$ we can first construct $\text{ PRVD}\!(\mathcal{P})$ and then we can traverse it to find the vertex of maximum distance. Both steps can be done in *Θ (n)* time resulting in the following.

#### Theorem 33

The Brocard angle of a convex polygon $\mathcal{P}$ can be found in *Θ (n)* time.

Following, we give tight bounds on the value of the Brocard angle.

#### Proposition 34

Given a convex polygon $\mathcal{P}$, the range of values of the Brocard angle is $(0,\pi /2 - \pi /n]$.

#### Proof

A $\pi /2 - \pi /n$ upper bound on the Brocard angle is given in [7, 16]. Such an angle is realized by regular polygons. The last illuminated point is the center of the polygon, which is simultaneously illuminated by all the floodlights at an angle of $\pi /2 - \pi /n$.

To prove the lower bound note that, while preserving convexity, we can smoothly transform a regular polygon into a polygon whose bounding box has width *w*, height *h*, and an aspect ratio *h*/*w* arbitrarily close to zero, so that $\alpha ^*$ is also arbitrarily close to zero. Hence it is possible to get any Brocard angle in the range $(0,\pi /2 - \pi /n]$. *□ *

**Illumination of a convex polygon by 3 floodlights**. Note that the three floodlights which realize $\alpha ^*$ suffice to illuminate $\mathcal{P}$; see the example of Fig. 29b.

#### Remark 35

A convex polygon $\mathcal{P}$ can be entirely illuminated by three $\alpha ^*$-floodlights.

Consider the following question [33]: given a convex polygon $\mathcal{P}$ with *n* vertices, what is the minimum angle $\beta ^*$, such that three *vertex* $\beta ^*$*-floodlights* (floodlights with their apices on vertices but not necessarily aligned with the edges) illuminate $\mathcal{P}$. A $\beta ^*=\pi /6$ solution for *n=3*, and a $\beta ^*= \pi /4$ solution for *n=4*, is given in [11]. For *n≥ 5*, the best bound is given by a generic $\beta ^*=\pi /3$ solution in [43].

Combining the results from Theorem 33, Proposition 34, and Remark 35 we can immediately derive the following.

#### Proposition 36

A convex polygon $\mathcal{P}$ with *n* vertices can be illuminated by three floodlights of aperture $\pi /2 - \pi /n$. These can be found in *Θ (n)* time.

This matches the current best results for this problem for $n\in \{3,4,6\}$. Further, it improves the best known result for *n=5*, from *π /3* to $\beta ^*=3\pi /10$. Further, this also positively answers the conjecture stated in [11] that any convex *n*-gon can be illuminated by three edge-aligned floodlights, each of them of angle $\pi /2 - \pi /n$.

## Rotating Rays Voronoi Diagram Restricted to Curves

Floodlight illumination problems have also been considered restricted to curves, see e.g., [12, 17, 23, 41]. Motivated by such problems, let $\mathcal{S}$ be a set of *n* rays in $\mathbb {R}^2$, and let the domain of interest be a simple curve $\mathcal{C}$. We denote by $\text{ RVD}_\mathcal{C}(\mathcal{S})$ the rotating rays Voronoi diagram of $\mathcal{S}$ restricted to $\mathcal{C}$. We show that $\text{ RVD}_\mathcal{C}(\mathcal{S})$ can be viewed as the lower envelope of distance functions in 2-space.

### Brocard Illumination of a Line

**Fig. 30:**
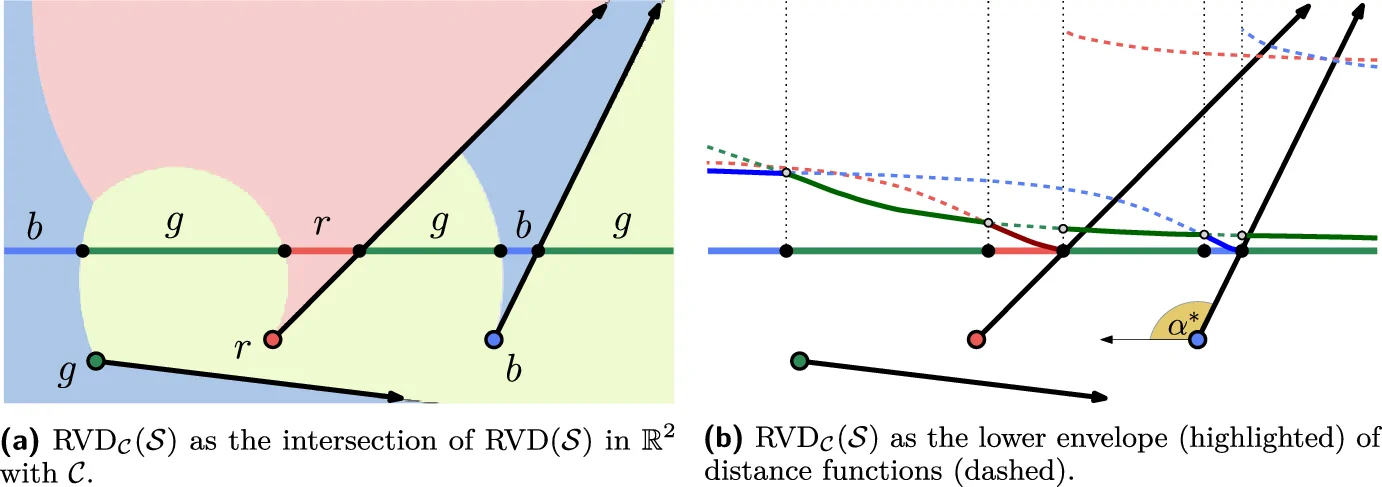
The curve $\mathcal{C}$ is the horizontal line $x_2=0$, and $\mathcal{S}$ is a set of 3 rays $\{r,b,g\}$

We first consider the curve $\mathcal{C}$ to be a line; see Fig. 30. We prove the following.

#### Theorem 37

Given a a set of *n* rays $\mathcal{S}$ and a line $\mathcal{C}$, $\text{ RVD}_\mathcal{C}(\mathcal{S})$ has complexity $O(n 2^{\alpha (n)})$ and it can be constructed in $O(n\alpha (n)\log n)$ time.

#### Proof

Without loss of generality, let $\mathcal{C}$ be the horizontal line $x_2=0$; see Fig. 30. Each site $r \in \mathcal{S}$ induces a distance function in 2-space which maps a point $x = (x_1,0) \in \mathcal{C}$ to point $x_{map} = (x_1,d_{\angle }(x,r))$ (dashed curves in Fig. 30b). Observe that if a ray *r* intersects $\mathcal{C}$ at point (*i*, 0), there is a point of discontinuity, and the distance function is split into two partially defined functions, one with domain up to *i* and one with domain starting at *i*. The diagram $\text{ RVD}_\mathcal{C}(\mathcal{S})$ is the lower envelope of all these distance functions projected down to $\mathcal{C}$. The lower envelope of *n* partially defined functions, where each pair of functions intersects at most *s* times, has $O(\lambda _{s+2}(n))$ complexity [19] and it can be constructed in $O(\lambda _{s+1}(n)\log n)$ time [21], where $\lambda _{s}(n)$ is the length of the longest (*n*, *s*) *Davenport-Schinzel sequence*.

Observe that the number of intersections of two distance functions is the same as the number of intersection of their bisecting circle with $\mathcal{C}$. In our case, a pair of functions intersects at most twice, as $\mathcal{C}$ may intersect twice the bisecting circle of the two respective rays, so *s=2*. Further, we have at most 2*n* partially defined functions. Thus, $\text{ RVD}_\mathcal{C}(\mathcal{S})$ has complexity $O(n 2^{\alpha (n)})$ and it can be constructed in $O(n\alpha (n) \log n)$ time, where *α (n)* is the inverse Ackermann function. *□ *

Considering the illumination of a line $\mathcal{C}$, given $\mathcal{S}$, the Brocard angle $\alpha ^*$, is realized at a vertex of $\text{ RVD}_\mathcal{C}(\mathcal{S})$, or at a point of $\mathcal{C}$ at infinity; see, e.g., in Fig. 30b, point *(-∞ ,0)* first illuminated by ray *b*. So, a simple traversal of $\text{ RVD}_\mathcal{C}(\mathcal{S})$ reveals $\alpha ^*$ in linear additional time.

### Brocard Illumination of a Closed Curve

The aforementioned approach can be generalized to arbitrary simple curves, both bounded and unbounded. We first consider $\mathcal{C}$ to be a closed convex curve, aiming to illuminate the interior of $\mathcal{C}$, i.e., the apices of the rays lie inside $\mathcal{C}$.

#### Theorem 38

Let $\mathcal{C}$ be a closed convex curve, and let the apices of the rays in $\mathcal{S}$ lie in the interior of $\mathcal{C}$. Then, $\text{ RVD}_\mathcal{C}(\mathcal{S})$ has complexity $O(\lambda _{s+2}(n))$ and can be constructed in $O(\lambda _{s+1}(n)\log n)$ time, where *s* is the maximum number of times $\mathcal{C}$ is intersected by a (bisecting) circle.

#### Proof

Assume that the curve $\mathcal{C}$ is parametrized in the following form, $\mathcal{C}: [0,1] \rightarrow \mathbb {R}^2$ with $\mathcal{C}(0) = \mathcal{C}(1)$. Analogously to the approach of Theorem 37, each site *r* induces a distance function on the curve $\mathcal{C}$, which maps a value *t ∈ [0,1]* to the point $(t,d_{\angle }(\mathcal{C}(t),r))$, and the result immediately follows the results of the envelopes of distance functions in 2-space [19, 21]. *□ *

**Fig. 31:**
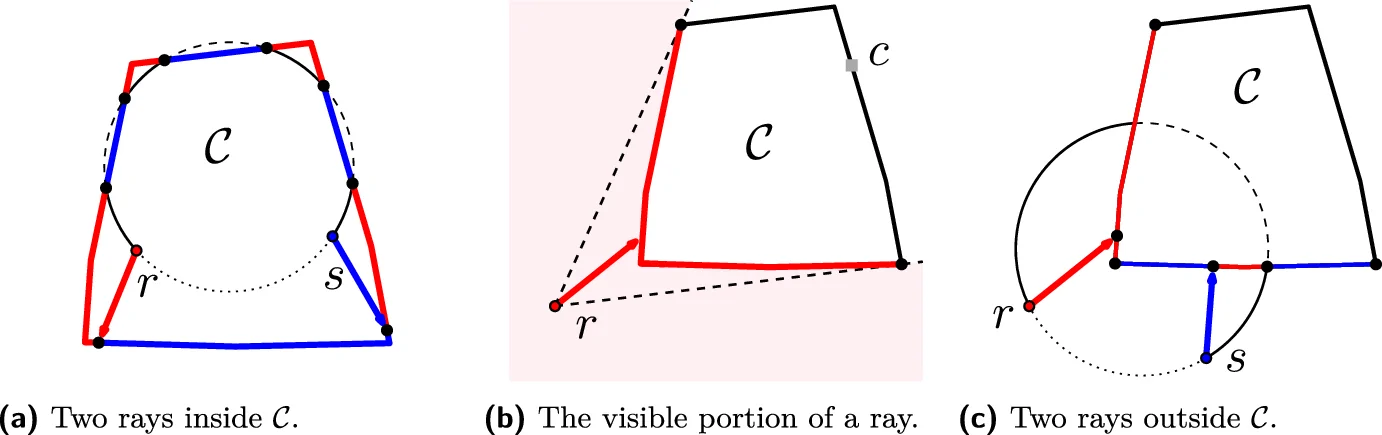
$\mathcal{C}$ is a convex polygon. The dominance regions of the rays along $\mathcal{C}$ are highlighted

As a corollary, if $\mathcal{C}$ is a circle, then $\text{ RVD}_\mathcal{C}(\mathcal{S})$ has complexity $O(n 2^{\alpha (n)})$ and it can be constructed in $O(n\alpha (n)\log n)$ time, since $\mathcal{C}$ intersects a bisecting circle at most twice; hence, *s=2*. On the contrary, if $\mathcal{C}$ is an *m*-sided convex polygon, then $\text{ RVD}_\mathcal{C}(\mathcal{S})$ has complexity $O(\lambda _{2m+2}(n))$ and it can be constructed in $O(\lambda _{2m+1}(n)\log n)$ time, since $\mathcal{C}$ intersects a bisecting circle at most 2*m* times, hence *s=2m*; see, e.g., the polygon in Fig. 31a.

If we consider the case of illuminating the exterior of a closed convex curve $\mathcal{C}$, i.e., the rays lie outside $\mathcal{C}$, we obtain better results. In this case, only a part of the curve $\mathcal{C}$ is *visible* by each input ray, where a point $x\in \mathcal{C}$ is visible by a ray *r* if the open line segment $\overline{p(r)x}$ does not intersect $\mathcal{C}$; see, e.g., in Fig. 31b, the visible portion of a ray *r* (point *c* is not visible by *r*). If a point *x* is not visible by a ray $r \in \mathcal{S}$, we set $d_{\angle }(x,r) = +\infty$.

#### Theorem 39

Let $\mathcal{C}$ be a closed convex curve, and let the apices of the rays in $\mathcal{S}$ lie outside $\mathcal{C}$. Then, $\text{ RVD}_\mathcal{C}(\mathcal{S})$ with visibility restrictions has complexity $O(n 2^{\alpha (n)})$ and it can be constructed in $O(n\alpha (n)\log n)$ time.

#### Proof

We apply the same approach used as in Theorem 37 and 38. To get the claimed results we show the part of the curve visible by any two rays is intersected at most twice (*s=2*) by a bisecting circle. See Fig. 31c.

**Fig. 32:**
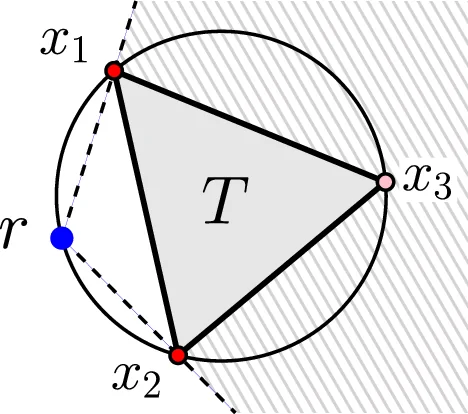
Illustration for the proof of Theorem 39. Point $x_3$ is not visbile from ray *r*. Triangle *T* is a subset of $\mathcal{C}$

Given a ray $r \in \mathcal{S}$ consider the portion of $\mathcal{C}$ visible by *r*. Suppose, for the sake of contradiction, that a bisecting circle defined by *r* intersects the visible part of $\mathcal{C}$ in at least 3 points $x_1,x_2,x_3$; refer also to Fig. 32. By definition, *p(r)* lies on the bisecting circle, so *p(r)* lies on one of the circular arcs $\overline{x_1x_2}$, $\overline{x_2x_3}$, or $\overline{x_3x_1}$; without loss of generality, suppose $p(r) \in \overline{x_1x_2}$. By the assumption *p(r)* lies outside $\mathcal{C}$, so obviously points $x_1,x_2 \in C$ obstruct the visibility of *r*, and $x_3$ is not visible by *r*. But $x_3$ was the intersection point of the bisecting circle with the visible portion of $\mathcal{C}$, a contradiction.

The part of $\mathcal{C}$ visible by two rays is a subset of the part visible by each of the rays independently, so it is intersected by a bisecting circle at most twice. Hence, *s=2* and as in Theorem 37 and 38 the combinatorial and algorithmic results follow. *□ *

**Fig. 33:**
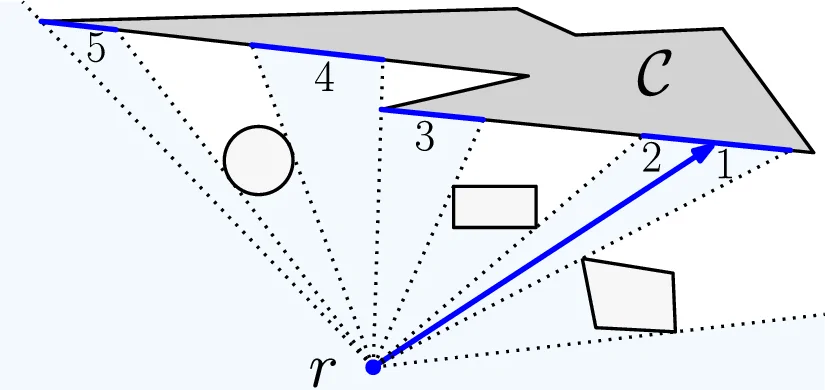
Illumination of $\mathcal{C}$ and the parameter *k*. The distance function of a ray *r* is split into 5 partial functions (k=5) by different types of breakpoints

**Extensions to other classes of curves**. Our approach can be extended to illuminate classes of curves which induce visibility restrictions to the rays/sites. As an example, refer to Fig. 33, and consider the illumination of a non-convex polygon $\mathcal{C}$ in the presence of other curves (*obstacles*). Given a ray *r*, the portion of $\mathcal{C}$ visible by *r* can be split into many maximal connected components, due to the visibility constraints; a split might be induced by the curve $\mathcal{C}$ itself (breakpoint (3-4) in Fig. 33); it can be induced by other curves (breakpoint (2-3) in Fig. 33); or it can be induced by the ray *r* itself (breakpoint (1-2) in Fig. 33).

If the part of the curve $\mathcal{C}$ visible by a ray $r_i$ is split in $k_i$ connected components, this implies that the corresponding distance function of $r_i$ is split into $k_i$ partially defined functions. Let $K = \sum _{i \in n} k_i$ be the total number of partially defined functions. Using results on lower envelopes of distance functions in 2-space [19, 21], we can derive that $\text{ RVD}_\mathcal{C}(\mathcal{S})$ has complexity $O(\lambda _{s+2}(K))$ and it can be constructed in $O(\lambda _{s+1}(K)\log K)$ time.

## Concluding Remarks

In this work, we studied a new Voronoi structure, the rotating rays Voronoi diagram. Our motivation for studying this diagram originates from the Brocard illumination problem in polygons. We exhibited a general method for solving the Brocard illumination problem in different domains: given a domain *D* and a set of rays $\mathcal{S}$, we can find the minimum angle $\alpha ^*$ needed to illuminate *D* using $\alpha ^*$-floodlights aligned with $\mathcal{S}$, by constructing $\text{ RVD}\!(\mathcal{S})$ restricted to *D*.

There are many interesting questions to investigate further, both related to the study of $\text{ RVD}\!(\mathcal{S})$ and to floodlight illumination problems. Regarding the $\text{ RVD}\!(\mathcal{S})$ in $\mathbb {R}^2$, we would like to settle whether the worst case combinatorial complexity is $\Theta (n^2)$ and whether it can be constructed in $o(n^{2 + \epsilon })$ time. Regarding the Brocard illumination of polygons, we would like to see how our approach could be extended to other classes of (non convex) polygons.

## Data Availability

No datasets were generated or analysed during the current study.

## Footnotes

1 The *O*(*n*) time analysis of the algorithm for convex polygons stated in [2] is not correct.

2 The disk diagram does not necessarily cover $\mathbb {R}^2$, for *n≥ 3*. This is because there are areas having a cyclic dominance relation among some sites; see for example the white/uncolored region in Fig. 18b.

3 A cyclic quadrilateral or inscribed quadrilateral is a quadrilateral whose vertices lie on a single circle, and therefore, the four perpendicular bisectors (of the sides) are concurrent. Also, a convex quadrilateral is cyclic if and only if its opposite angles are supplementary (i.e., their sum is *π *).
